# A manufacturer-aware cross-system benchmark for pediatric pharmacovigilance signal concordance

**DOI:** 10.3389/fphar.2026.1900271

**Published:** 2026-09-07

**Authors:** Jinwan Shi, Jieting Xia, Huanqiong Li, Shuyan Zhang, Bowen Shi

**Affiliations:** 1 Foshan Sanshui District People’s Hospital, Foshan, Guangdong, China; 2 School of Medical Information Engineering, Guangdong Pharmaceutical University, Guangzhou, Guangdong, China

**Keywords:** adverse drug reactions, benchmark, Canada Vigilance, disproportionality analysis, FAERS, JADER, manufacturer cross-filing, pediatric pharmacovigilance

## Abstract

**Introduction:**

Pediatric adverse drug reactions are difficult to evaluate after approval because pediatric trial evidence is limited and spontaneous-report systems differ in product naming, event coding, seriousness fields, and duplicate submissions.

**Methods:**

We developed a manufacturer-aware benchmark for pediatric pharmacovigilance signal concordance using the FDA Adverse Event Reporting System (FAERS)/AERS as the source system, Canada Vigilance as a cross-system reporting-signal comparator, and JADER for descriptive third-country context. Active ingredients and five clinically adjudicated serious endpoint families were harmonized before detectable manufacturer cross-filing was screened.

**Results:**

The supervised benchmark contained 628 drug–endpoint–period rows: 147 manufacturer-screened Canada Vigilance reporting-signal positives and 481 Canada Vigilance co-report-absent comparators. An endpoint-conditioned FAERS co-reporting comparator achieved an area under the precision–recall curve (AUPRC) of 0.753. Elastic-net logistic regression reached 0.767, and a LightGBM comparator reached 0.774, but clustered paired-difference intervals *versus* the conditioned co-report comparator included zero. In 30 repeated drug-held-out partitions, chemical structure increased the mean AUPRC from 0.764 to 0.796, but the corresponding increment was –0.003 under unseen-scaffold partitions; target and pathway indicators produced no further material increase. Future-period support was concentrated in anaphylaxis: 40 drug pairs remained after manufacturer screening, including 37 with FAERS source exposure, and four of five FAERS scores ranked supported pairs above permutation expectations.

**Discussion:**

Clinical label specificity, detectable duplicate screening, and fair source-signal comparators materially influenced model evaluation. The resulting resource provides a reproducible reference for pediatric signal triage and for testing whether new pharmacological features or models add information beyond established reporting statistics.

## 1 Introduction

Children remain one of the most difficult populations for evaluating the safety of medicines after approval. Developmental changes in the pharmacokinetics, pharmacodynamics, immunity, organ maturation, and patterns of use mean that adverse drug reactions (ADRs) observed in adults cannot be assumed to transfer directly to pediatric patients. At the same time, many medicines used in children are supported by weaker and more indirect evidence than that in adults, and off-label-use remains common across pediatric care (van der Zanden et al., 2022; Black et al., 2015). When serious pediatric drug–event patterns emerge in routine use, regulators, clinicians, and researchers need methods that recognize them early, compare them across public data sources, and distinguish reproducible reporting patterns from database-specific artifacts. Systematic reviews continue to show that pediatric ADRs and drug-related hospital admissions contribute to the clinical burden, while estimates vary substantially with definitions, ascertainment strategies, and denominators (Smyth et al., 2012; Eberl et al., 2024; Ramos et al., 2024).

Public spontaneous-reporting systems are central to the task because they capture suspected drug-event reports at a scale unavailable to trials and many pediatric observational cohorts. FDA Adverse Event Reporting System (FAERS)/AERS, Canada Vigilance, and Japanese Adverse Drug Event Report (JADER) each provide large public windows into post-marketing safety reporting (U.S. Food and Drug Administration, 2026; Health Canada, 2026; Pharmaceuticals and Medical Devices Agency, 2026). Conventional proportional reporting ratios, reporting odds ratios, and information-component approaches remain widely used because they are transparent and reproducible from individual case-safety reports (Evans et al., 2001; van Puijenbroek et al., 2002; Bate et al., 1998; Jiao et al., 2024). Yet, the scale does not create a reliable benchmark by itself. Public reporting systems differ in drug naming, report structure, seriousness fields, age coding, reporter composition, duplicate submissions, and event vocabularies. Recent reporting guidance, therefore, emphasizes provenance, case definitions, comparator choice, duplicate handling, and cautious interpretation of disproportionality analyses (Fusaroli et al., 2024a; Fusaroli et al., 2024b). These requirements are especially consequential in pediatrics, where sparse endpoint support, age-specific terminology, vaccine-heavy reporting patterns, and seriousness definitions can materially change the apparent signal support.

The missing resource is a clinically adjudicated, cross-system benchmark that makes these public data comparable while preserving source-specific schema differences. Existing reporting-system reviews and FAERS-focused preprocessing work have described persistent problems in drug normalization, duplicate records, and database-specific extraction choices (Bailey et al., 2016; Alomar et al., 2020; Giunchi et al., 2023; Parry et al., 2025). Duplicate reports remain a recurring pharmacovigilance challenge, including reports introduced through manufacturer- and literature-reporting pathways (Kiguba et al., 2024). Pediatric benchmark construction adds a second layer of difficulty. Endpoint definitions must exclude disease manifestations, laboratory-only findings, congenital conditions, and indication codes while retaining serious pediatric ADR phenotypes. Pediatric reference sets and pediatric drug-effect resources have shown the value of pediatric-specific signal discovery and performance testing (Osokogu et al., 2015; Giangreco and Tatonetti, 2022; Tian et al., 2026). A cross-system public benchmark, therefore, requires active-ingredient-level product harmonization, pediatric endpoint adjudication, and detectable manufacturer cross-filing control before reporting-signal concordance is evaluated.

This benchmark gap is equally important for machine-learning claims and for conventional pharmacovigilance. Knowledge-graph and machine-learning approaches are increasingly proposed for adverse-event prediction and signal detection, but their apparent value depends on the labels, comparators, and leakage controls used for evaluation (Hauben et al., 2024; Farnoush et al., 2024; Tian et al., 2026). In spontaneous-report settings, models may exploit endpoint prevalence, repeated drug identities, or the reporting volume, producing optimistic estimates of cross-system concordance. Transparent source-signal comparators can remain difficult to outperform once endpoint prevalence, period structure, and duplicate handling are explicit. A useful pediatric benchmark, therefore, needs second-system target labels, strong pharmacovigilance reference comparators, and direct tests of whether richer drug features add reproducible information beyond source reporting signals.

Here, we construct and evaluate a manufacturer-aware, cross-system benchmark for pediatric pharmacovigilance signal concordance. FAERS/AERS, Canada Vigilance, and JADER were assigned distinct roles. Medicinal products were harmonized to active-ingredient identities using public vocabularies and chemical backbones (MedDRA MSSO, 2026; U.S. National Library of Medicine, 2026). Five serious pediatric endpoint families were clinically adjudicated using MedDRA preferred terms, and detectable manufacturer-number cross-filing was screened before drug–event–period labels were defined. Reporting-signal comparators, interpretable models, boosted-tree baselines, and added drug-feature families were evaluated under common source-side feature rules. Evaluation included clustered uncertainty, drug-held-out partitions, and label-shuffle controls. Anaphylaxis was examined as a future-period source-ranking enrichment task. JADER provided descriptive third-country context because its public schema lacks a comparable report-level seriousness field. The resulting benchmark supports evaluation of pediatric drug-safety signal concordance and added drug-context features.

## 2 Materials and methods

### 2.1 Study design and reporting framework

We constructed a retrospective public-data benchmark of concordance between pediatric adverse-event signals in the FAERS and manufacturer-screened support in Canada Vigilance. Spontaneous-report systems lack exposed-person denominators; therefore, source summaries are counts or report proportions rather than incidence estimates. Reporting followed STROBE, RECORD, and READUS-PV guidance (von Elm et al., 2007; Benchimol et al., 2015; Fusaroli et al., 2024a; Fusaroli et al., 2024b).

**FIGURE 1:**
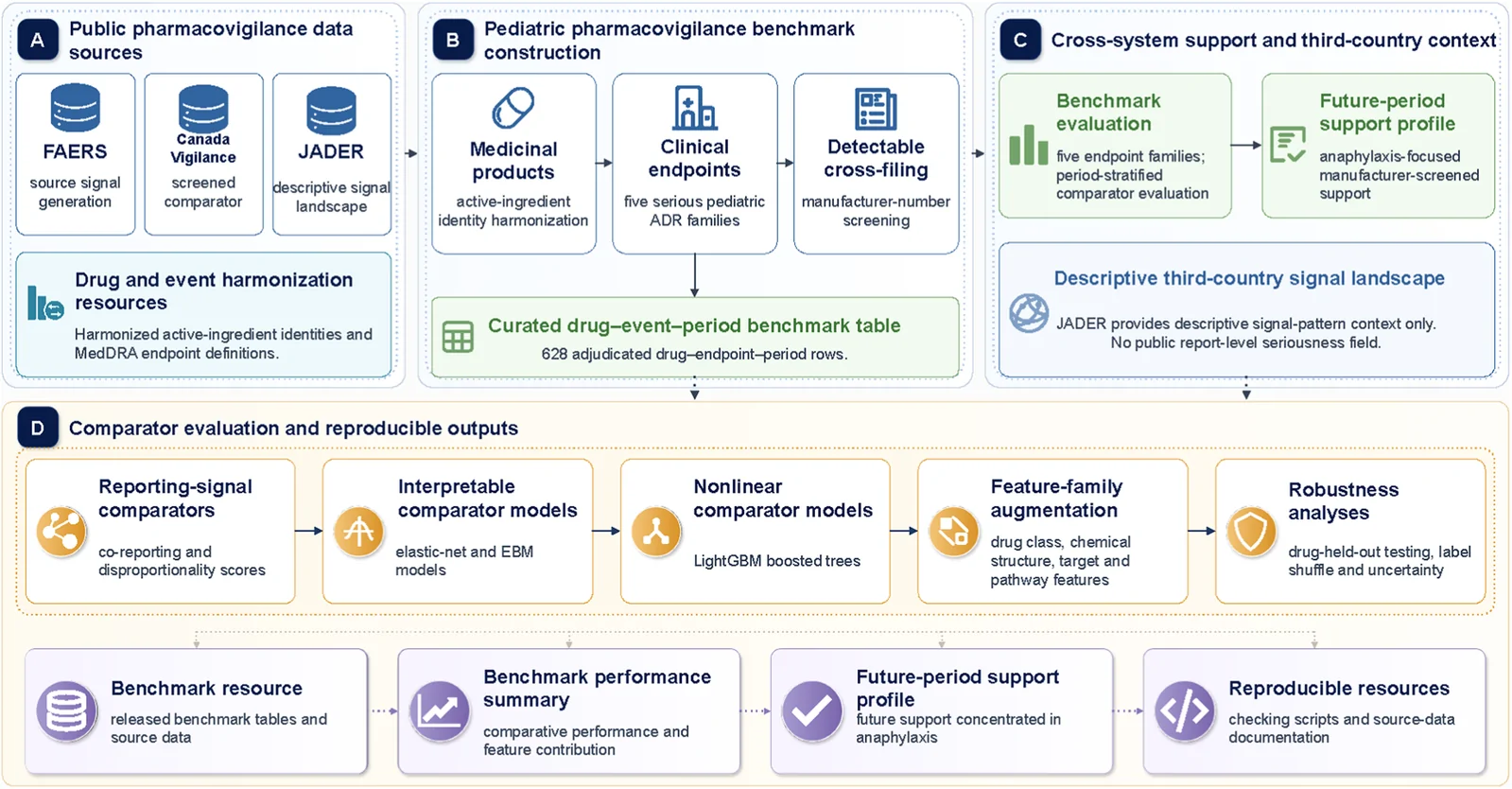
Study framework for a manufacturer-aware pediatric pharmacovigilance benchmark. **(A)** Public sources and drug and event harmonization resources. **(B)** Construction of drug–endpoint–period rows from medicinal-product identity, clinical endpoints, and detectable cross-filing. **(C)** Cross-system support and descriptive third-country context. **(D)** Comparator evaluation, robustness analyses, and reproducible outputs.

The workflow comprised source harmonization, clinical adjudication of five serious pediatric endpoint families, screening of detectable manufacturer cross-filing, and comparator evaluation under common labels and splits (Figure 1; Supplementary Figure S8). A separate anaphylaxis analysis tested whether FAERS scores ranked future Canada-supported drug pairs above a permutation null.

### 2.2 Public pharmacovigilance sources

FAERS supplied source signals; Canada Vigilance supplied the manufacturer-screened comparator because its public extract includes report-level seriousness and manufacturer authorization-holder identifiers (U.S. Food and Drug Administration, 2026; Health Canada, 2026). JADER contributed descriptive third-country context only because its public schema lacks a comparable report-level seriousness field (Pharmaceuticals and Medical Devices Agency, 2026). Source roles and field differences are summarized in Supplementary Table S2.

The 89 FAERS/AERS quarterly demographic tables reconciled to 24,389,966 report rows. Pediatric eligibility in FAERS/AERS and Canada Vigilance required an age convertible to <18 years; standard unit conversions were applied, while missing, inconsistent, or non-convertible ages were excluded from pediatric labels. Seriousness was defined from each source’s public seriousness or outcome fields. JADER age categories were retained without conversion, and incomplete years were excluded from longitudinal plots.

The exact numeric age was unavailable in 42.0% of FAERS/AERS reports and 23.0% of Canada Vigilance reports (Supplementary Table S17).

We compared age-complete and age-missing reports by seriousness, sex, reporter, report type, calendar period, manufacturer-identifier availability, and the 20 most frequently suspected ingredients and reaction terms; standardized differences and Jaccard overlap are reported in Supplementary Table S21. Age-missing reports were not entered in benchmark labels or model training.

### 2.3 Medicinal-product identity harmonization

Product strings were normalized to study-level medicinal-product identities before label construction. RxNorm ingredient relations aligned salts, precise ingredients, brands, and formulations to base ingredients (Nelson et al., 2011; U.S. National Library of Medicine, 2026); DrugCentral and ChEMBL supported international names and structure evidence (Ursu et al., 2017; Mendez et al., 2019). Prodrugs, metabolites, stereoisomers, and ambiguous names remained distinct, unless identity was independently confirmed. Unresolved products remained in descriptive accounting but were excluded from structure-dependent analyses (Supplementary Table S9).

### 2.4 Endpoint definition and MedDRA adjudication

Five endpoint families were selected before model evaluation: anaphylaxis, acute kidney injury, hepatic injury, QT/proarrhythmia, and seizure (Supplementary Table S12). Selection required clinical seriousness, pharmacological plausibility, a specific MedDRA phenotype, and sufficient cross-system representation. Clinical guidance defined the phenotype scope (Cardona et al., 2020; Kidney Disease: Improving Global Outcomes Acute Kidney Injury Work Group, 2012; Sethi et al., 2021; Fontana et al., 2023; U.S. Food and Drug Administration, 2022; Khatib et al., 2021; Beniczky et al., 2025); term membership was adjudicated independently. These families are not an exhaustive catalog of serious pediatric ADRs.

Candidate terms were mapped to MedDRA version 28.0 preferred-term and hierarchy files and assigned as primary, sensitivity-only, or excluded (MedDRA MSSO, 2026). Primary terms represented the endpoint itself rather than laboratory findings, congenital or infectious conditions, pregnancy/fetal conditions, indications, non-specific symptoms, or third-party events. One retained candidate, atypical absence seizure, entered the preferred-term hierarchy in version 28.1 but did not occur through 2024Q4. An audit through version 29.0 identified no change in terminology affecting the benchmark labels; future changes will undergo the same adjudication before inclusion.

Two adjudicators trained in pharmacovigilance, pediatric medication safety, and clinical terminology independently classified candidate terms; agreement ranged from Cohen’s κ=0.74 to 1.00. A third adjudicator resolved disagreements using the hierarchy, endpoint definitions, and pediatric plausibility, without reference to the sample size. Acute kidney injury had the lowest retained-endpoint agreement, and its endpoint-specific estimates were treated as descriptive. Final term lists, decisions, rationales, and boundary sensitivities are reported in Supplementary Tables S1, S7, and S14–S16.

### 2.5 Detectable cross-filing and duplicate screening

FAERS manufacturer report numbers and Canada authorization holder numbers were normalized to uppercase alphanumeric keys and matched exactly. Matching keys identified detectable cross-filing and were removed from Canada support counts; missing keys were unscreenable, and no fuzzy company linkage was inferred. Public extracts lack narratives and complete case identifiers, so this procedure cannot identify all duplicate reports (Supplementary Table S8).

For anaphylaxis, a sensitivity screen additionally required agreement on the exact date, medicinal-product identity, preferred term, sex, and pediatric age-band among non-manufacturer-matched reports. A wider window of dates remained descriptive because chance matching was plausible.

### 2.6 Drug-event-period benchmark labels

The benchmark unit was a medicinal-product identity, endpoint family, and period. Historical rows spanned 2014Q3–2020Q4 and future rows 2021Q1–2024Q4. The historical start followed sustained identity-mapping coverage above the pre-specified 80% floor; the future boundary aligned with the pre-2021 cut-off for dated pharmacological features (Supplementary Table S20). A wider 2012Q3 start yielded the same 40 manufacturer-screened anaphylaxis pairs (Supplementary Table S8). Entry required FAERS source exposure, and Canada Vigilance defined the target after detectable cross-filing was removed.

FAERS source signals and Canada Vigilance target signals were derived from conventional disproportionality criteria. A disproportionality signal denotes drug-event co-reporting that is higher than expected from the surrounding database; it is a screening statistic rather than evidence of causality. For each drug-event-period cell, we computed co-report counts, reporting odds ratio lower-confidence bounds, proportional reporting ratios, chi-square statistics, and an information-component-like score (Evans et al., 2001; van Puijenbroek et al., 2002; Bate et al., 1998). For the 2×2 report table, a denoted reports with both product and endpoint, b denoted product reports without the endpoint, c denoted endpoint reports without the product, and d denoted reports with neither. Let $a^{\prime }=a+0.5$ , $b^{\prime }=b+0.5$ , $c^{\prime }=c+0.5$ , $d^{\prime }=d+0.5$ , and n=a+b+c+d . The signal metrics were calculated as

$$\begin{array}{cccc} ROR & =\frac{a^{\prime }d^{\prime }}{b^{\prime }c^{\prime }}, & {ROR}_{\mathrm{L95}} & =\exp\;\left[\log\left(ROR\right)-1.96\sqrt{\frac{1}{a^{\prime }}+\frac{1}{b^{\prime }}+\frac{1}{c^{\prime }}+\frac{1}{d^{\prime }}}\right],\\ PRR & =\frac{a^{\prime }/\left(a^{\prime }+b^{\prime }\right)}{c^{\prime }/\left(c^{\prime }+d^{\prime }\right)}, & \chi^{2} & =\frac{n{\left(ad-bc\right)}^{2}}{\left(a+b\right)\left(c+d\right)\left(a+c\right)\left(b+d\right)},\\ E & =\frac{\left(a+b\right)\left(a+c\right)}{n}, & {IC}_{\text{like}} & =\log_{2}\;\left(\frac{a+0.5}{E+0.5}\right). \end{array}$$

The chi-square statistic was set to zero if its denominator was zero. Supplementary Table S19 provides the corresponding formulas, symbols, and interpretations.

A binary signal required at least three co-reports and agreement between at least two of three families: ${ROR}_{\mathrm{L95}}>1$ , PRR≥2 with $\chi^{2}>4$ , or ${IC}_{\text{like}}>0$ . The report minimum excluded sparse singleton and doubleton co-reports, and agreement reduced dependence on any one metric. Canada Vigilance positives met the same signal rule after manufacturer screening. FAERS-exposed rows with zero Canada Vigilance co-reports were comparators; rows with one or two were borderline (Supplementary Tables S3 and S4).

After the 18-row anaphylaxis future-period enrichment set was reserved, 803 drug–endpoint–period rows remained for label accounting. The reserved anaphylaxis set was disjoint from the supervised benchmark, so no drug–endpoint–period row contributed to both the pooled supervised metrics and the anaphylaxis enrichment analysis. The primary supervised benchmark contained 628 rows: 147 manufacturer-screened Canada Vigilance reporting-signal positives and 481 Canada Vigilance co-report-absent comparator rows. A further 155 borderline rows and 20 ambiguous rows were retained for accounting and sensitivity analyses but excluded from primary supervised metrics.

Sensitivity analyses varied co-report, signal-agreement, and Canada Vigilance -observability thresholds and assigned borderline rows to either class. Across 13 scenarios and 10 unique label universes, the original folds, features, and fold-specific settings were reused without retuning; paired AUPRC differences used 2,000 medicinal-product-clustered bootstrap replicates (Supplementary Table S23).

### 2.7 Benchmark splits and source-side features

Primary evaluation used five-fold cross-validation stratified by endpoint, period, and label. The future period contained too few positives for a powered five-endpoint temporal holdout, so future-period and endpoint-specific summaries were descriptive. Product identities were not predictors and could recur across the primary folds; drug-held-out partitions were used when the product class or structure was added.

The core predictors were five FAERS reporting statistics, endpoint family, and period. Preprocessing was fitted within training folds. Canada Vigilance targets, future-target information, manufacturer-screening flags, product identifiers, and post-cut-off adverse-event knowledge were excluded (Supplementary Table S5).

### 2.8 Comparator models and performance estimation

Comparators comprised prevalence baselines, raw and endpoint/period-conditioned FAERS score models, elastic-net logistic regression, an explainable boosting machine (EBM), and LightGBM (Pedregosa et al., 2011; Ke et al., 2017). Model selection and preprocessing were confined to training data; Supplementary Table S5 reports tuning and the random-forest specificity comparator.

The metrics were AUPRC, AUROC, Brier score, calibration intercept and slope, expected calibration error, fixed-budget precision, and top-decile enrichment. AUPRC was the primary metric because supported rows were a minority and the task prioritized review ranking. Definitions and formulas are provided in the glossary and Supplementary Table S19. Uncertainty and paired differences used medicinal-product-clustered bootstrap resampling; endpoint-specific comparisons used 5,000 replicates and were descriptive without multiplicity adjustment (Supplementary Table S24).

### 2.9 Feature-family augmentation analyses

Secondary analyses added ATC class, chemical structure, target, and pathway features to the FAERS baseline. Structures yielded radius-2, 1,024-bit Morgan fingerprints and seven physicochemical descriptors; target and Reactome indicators used ChEMBL and DrugCentral evidence available before 2021, where dates were present (WHO Collaborating Centre for Drug Statistics Methodology, 2026; Rogers and Hahn, 2010; RDKit Developers, 2026; Mendez et al., 2019; Ursu et al., 2017; Gillespie et al., 2022). Adverse-event databases and label-derived adverse-reaction features were excluded.

Repeated drug-held-out partitions tested unseen-product performance. The pre-specified +0.015 AUPRC difference was an exploratory materiality screen, not a clinical or statistical significance threshold; inference used medicinal-product-clustered intervals. A separate 30-partition analysis grouped 275 products by the Bemis–Murcko scaffold so neither identity nor scaffold crossed partitions. Within-partition permutation attributed increments to ECFP4, physicochemical, and ATC features; label shuffling provided a negative control (Supplementary Table S10).

Label efficiency was assessed at 25%, 50%, 75%, and 100% of training rows using fixed test folds and 30 stratified sub-samples below 100% (Supplementary Figure S7; Supplementary Table S13).

### 2.10 Anaphylaxis future-period support enrichment

Only anaphylaxis exceeded the pre-specified feasibility reference of 30 manufacturer-screened future Canada-supported drug pairs. This reference governed the analysis scope, not significance. The narrow four-term, vaccine-excluded definition yielded 40 supported pairs, 37 with FAERS exposure; exact-date screening retained 36 and 34, respectively (Supplementary Table S10).

Fixed FAERS scores ranked supported pairs among 1,638 source-exposed candidates. The rank percentiles and top-fraction capture were compared with 10,000 support-label permutations.

### 2.11 Descriptive JADER landscape

JADER reaction terms were matched exactly to MedDRA/J preferred terms. Because its public schema lacks a FAERS/Canada-comparable report-level seriousness field, JADER was restricted to descriptive signal-pattern context outside label, cross-filing, and future-support analyses.

### 2.12 Ethics, data availability, and software

Analyses used de-identified public regulatory extracts without narratives, medical records, private identifiers, recruitment, or intervention. Source data are available from FDA, Health Canada, and PMDA. Derived tables and documentation are archived on Zenodo (https://doi.org/10.5281/zenodo.21634673), with checking and summarization code on GitHub (https://github.com/BowenShiGDPU/Pediatric-pharmacovigilance-signal-concordance-benchmark). The release contents and controlled-vocabulary restrictions are summarized in Supplementary Table S6.

## 3 Results

### 3.1 A public-data framework for pediatric pharmacovigilance concordance

The study assembled three public spontaneous-reporting systems into distinct, non-interchangeable roles (Figure 1; Table 1). FAERS/AERS provided the source-side reporting signals, Canada Vigilance provided the manufacturer-screened reporting-signal comparator, and JADER provided a descriptive third-country signal landscape. The public extracts contained 24.39 million FAERS/AERS reports, 1.24 million Canada Vigilance reports, and 1.03 million JADER reports. The two North American sources contained report-level seriousness fields, while JADER lacked a comparable public report-level seriousness field. Serious pediatric endpoint construction and manufacturer-screened support analyses were, therefore, restricted to FAERS/AERS and Canada Vigilance, with JADER retained as contextual signal-pattern evidence. Additional source-landscape, demographic, schema-comparability, and drug-class summaries are provided in Supplementary Figures S1–S4.

**TABLE 1:** Public pharmacovigilance sources and analysis roles.

Source	Public extract size	Role in this study	Key public fields used	Main design boundary
FAERS/AERS	24.39 million reports	Source-side signal generation and United States report landscape	Seriousness/outcome fields; manufacturer report number present in 95.6% of reports; exact numeric age unavailable in 42.0%	Spontaneous reports lack exposed-person denominators; source scores are reporting signals, not incidence estimates
Canada Vigilance	1.24 million reports	Manufacturer-screened reporting-signal comparator for serious pediatric support	Public seriousness field; manufacturer authorization holder number present in 74.2% of reports; exact numeric age unavailable in 23.0%	Manufacturer screening detects only observable cross-filing in public fields
JADER	1.03 million reports	Descriptive third-country signal landscape	MedDRA/J preferred terms and public age/sex categories	Lacks a comparable public report-level seriousness field and manufacturer-control key; reserved for descriptive signal-pattern context

**FIGURE 2:**
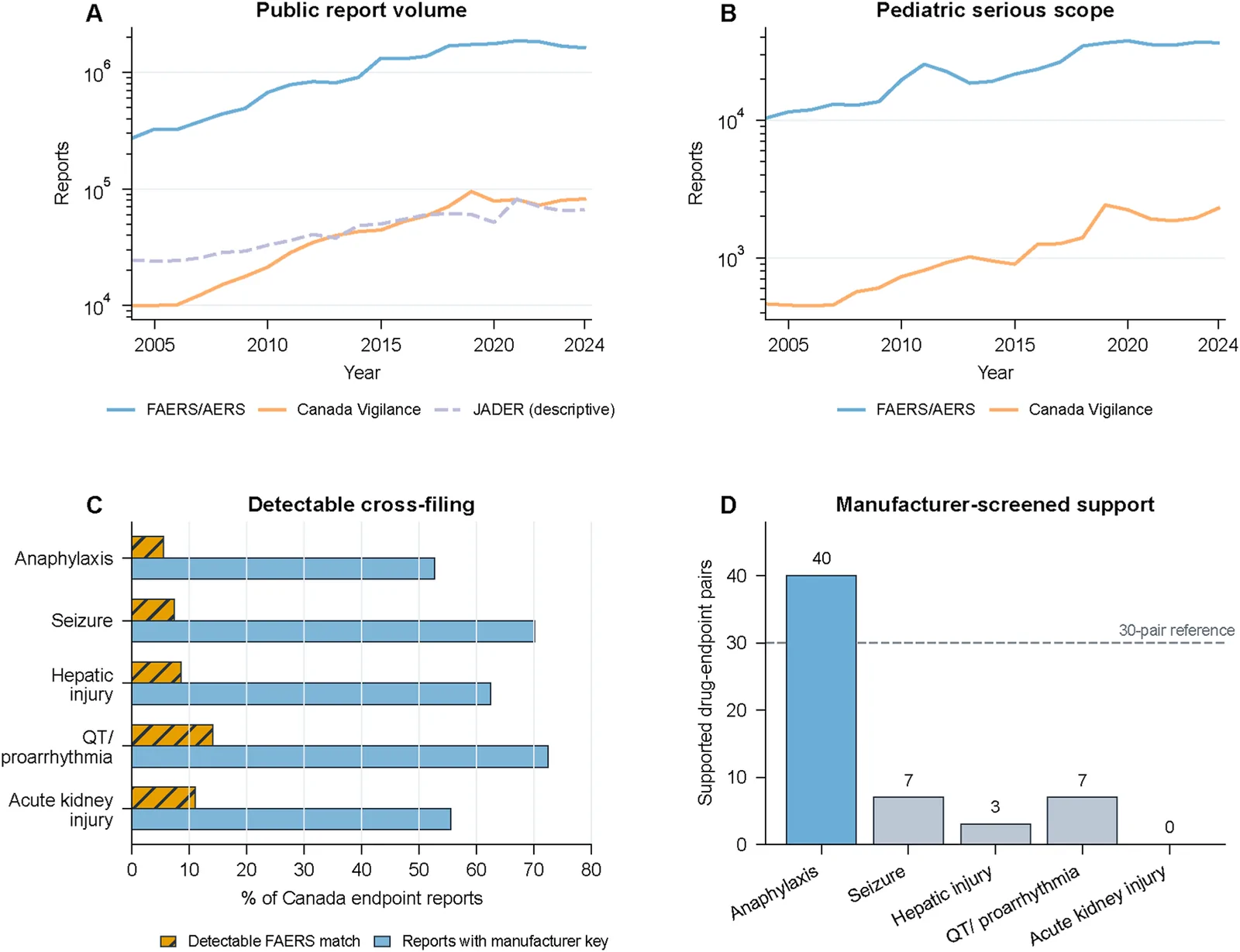
Public-reporting landscape, detectable cross-filing, and endpoint feasibility. **(A)** Annual public-report volumes. **(B)** Annual serious pediatric report counts in sources with comparable seriousness fields. **(C)** Manufacturer-key coverage and the fraction of report rows removed by exact-key cross-filing screening. **(D)** Manufacturer-screened future-period support by endpoint; the horizontal line marks the pre-specified 30-pair feasibility reference for a separate endpoint-level analysis. Counts are spontaneous-report counts, not incidence rates; JADER appears only in descriptive-source panels.

The public schemas also differed in duplicate-screening fields. Manufacturer-control keys were present in 95.6% of FAERS/AERS reports and 74.2% of Canada Vigilance reports, with no equivalent public key available in JADER. These fields supported exact-key screening of detectable manufacturer cross-filing before Canada Vigilance reporting-signal support was counted. In JADER, age and sex data were available through coarser public categories than in the North American sources. These differences in schema defined a harmonized cross-system concordance analysis based on the reporting counts (Figure 2; Supplementary Figures S1–S4).

Successive eligibility steps converted the harmonized source data into a defined set of drug–endpoint–period rows (Supplementary Figure S8; Supplementary Table S22). Of 18,635 candidate rows, 17,081 lacked the required FAERS source signal, 635 had insufficient Canada Vigilance observability, and 98 did not meet the primary medicinal-product identity criterion. Eighteen future-period anaphylaxis rows were reserved for the separate enrichment analysis. The remaining 803 rows entered benchmark label accounting.

Age completeness was associated with the reporting composition in both North American sources (Supplementary Table S21). Among FAERS/AERS reports, missing or unknown sex was recorded in 26.1% of age-missing reports and 2.1% of age-available reports; serious outcomes were recorded in 51.9% and 66.7% reports, respectively. Consumer reports accounted for 52.0% of age-missing reports and 37.4% of age-available reports. In Canada Vigilance, missing or unknown sex occurred in 18.1% and 1.3% reports, respectively. Manufacturer authorization-holder sources accounted for 79.3% and 63.5%, while hospital sources accounted for 2.9% and 11.9% reports. Sixteen of the 20 most frequent FAERS/AERS reaction terms and 17 of the 20 most frequent Canada Vigilance reaction terms were shared between age-completeness groups. Suspected-ingredient overlap was lower, at eight of 20 and 13 of 20. Age missingness was, therefore, associated with report provenance and medicinal-product mix.

### 3.2 Clinical endpoint adjudication produced a compact supervised benchmark

Five serious pediatric endpoint families entered the primary benchmark: acute kidney injury, anaphylaxis, hepatic injury, QT/proarrhythmia, and seizure (Table 2). Each represented a clinically serious pediatric ADR phenotype with pharmacological plausibility and a specific MedDRA preferred-term definition (Supplementary Table S12). Clinical adjudication retained 25 primary hepatic-injury terms after laboratory-only findings, and viral hepatitis terms, pregnancy/perinatal terms, and unrelated conditions were assigned to sensitivity-only or excluded categories. The seizure definition retained 25 primary terms and excluded febrile, metabolic, psychogenic, prophylaxis, and infectious-syndrome terms from the primary endpoint. Acute kidney injury used a three-term primary definition and had the lowest adjudicator agreement among the retained families (Supplementary Table S1).

**TABLE 2:** Endpoint definitions and primary benchmark label accounting.

Endpoint family	Primary endpoint scope after clinical adjudication	Supervised labels, positive/Canada co-report-absent	Borderline or ambiguous rows withheld	Definition note
Acute kidney injury	Three primary preferred terms	32/122	42	Consensus definition emphasizes clinically expressed acute kidney injury; laboratory-only, chronic, fetal, neonatal, and viral mimics are outside the primary label
Anaphylaxis	Four primary preferred terms	34/24	32	Restricted to anaphylactic or anaphylactoid reaction/shock; the separate support-enrichment set is disjoint from the supervised benchmark
Hepatic injury	25 primary preferred terms	17/156	46	Primary scope emphasizes clinical hepatic injury, cholestasis, failure, and drug-triggerable hepatic injury; laboratory-only, viral, neonatal/perinatal, chronic, and context-specific mimics are outside the primary label
QT/proarrhythmia	Six primary preferred terms	22/124	25	Primary scope includes QT prolongation, torsade de pointes, and ventricular arrhythmia; congenital long-QT and non-specific arrest/death terms are outside the primary label
Seizure	25 primary preferred terms	42/55	30	Primary scope covers organic seizure and status terms; febrile, metabolic, psychogenic, prophylaxis, and infectious-syndrome terms are outside the primary label
Total	Five endpoint families	147/481	175	Positives are manufacturer-screened Canada reporting-signal positives; 18 additional future anaphylaxis pairs were reserved for the separate support analysis and are not included in this column

The primary definitions produced 147 positives, 481 Canada Vigilance co-report-absent comparators, and 155 borderline rows. The endpoint-boundary sensitivity definitions produced 150, 481, and 157 rows, respectively (Supplementary Table S16). Preferred-term roles changed in all five families: primary-term counts increased for acute kidney injury, hepatic injury, and QT/proarrhythmia; were unchanged for anaphylaxis; and decreased by one for seizure. The three additional positives occurred in historical acute kidney injury. Thus, sensitivity-only terms contributed substantially to the report volume, particularly in hepatic and kidney injury, without materially changing the supervised label set (Supplementary Tables S14–S12).

After medicinal-product normalization, endpoint adjudication, source exposure requirements, and detectable manufacturer cross-filing removal, the supervised benchmark contained 628 drug–endpoint–period rows: 147 manufacturer-screened Canada Vigilance reporting-signal positives and 481 Canada Vigilance co-report-absent comparator rows. A further 155 borderline rows and 20 ambiguous rows were held out of primary supervised metrics. Positives were distributed unevenly across the five families: seizure contributed 42, anaphylaxis 34 after reserving the separate support set, acute kidney injury 32, QT/proarrhythmia 22, and hepatic injury 17. Primary evaluation was, therefore, pooled and stratified by endpoint and period; per-endpoint estimates were descriptive.

The relative performance of the fitted comparators was stable across alternative label and observability definitions (Supplementary Table S23). Thirteen specifications, representing 10 unique label universes, varied the minimum Canada Vigilance report count, required agreement among signal families, observability threshold, and treatment of borderline rows. Elastic-net differences from endpoint-conditioned co-reporting ranged from −0.015 to +0.025, with all clustered intervals including zero. LightGBM differences ranged from −0.043 to +0.045. Its clustered intervals generally included zero, and no monotonic advantage emerged from stricter or more permissive labels. These analyses supported the primary label definition without treating a single threshold choice as determinative.

The examples in Supplementary Table S11 trace the source-side signal strength, endpoint-specific target labels, future-period support, and detectable manufacturer screening through the benchmark rules.

Future-period support was concentrated in anaphylaxis. After manufacturer screening, anaphylaxis retained 40 supported future drug pairs, whereas the four other endpoint families retained 0–7 pairs each (Figure 2). Therefore, anaphylaxis was analyzed separately by source-ranking enrichment, while all five endpoint families contributed to the pooled benchmark.

### 3.3 Transparent source-signal comparators formed a strong benchmark

**FIGURE 3:**
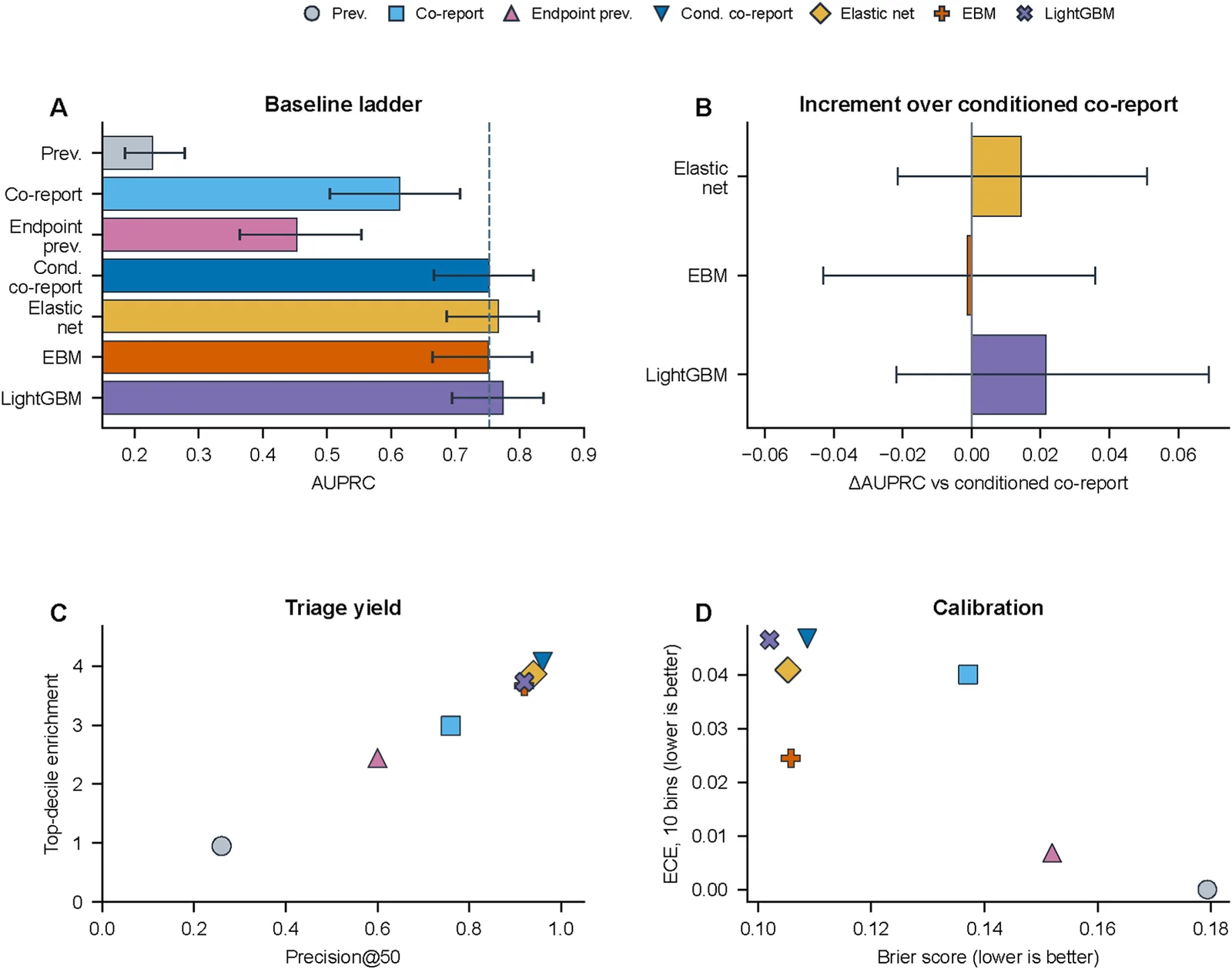
Benchmark comparator performance. **(A)** AUPRC estimates for prevalence (Prev.), raw co-reporting, endpoint/period prevalence (Endpoint prev.), endpoint-conditioned co-reporting, elastic-net logistic regression, explainable boosting machine (EBM), and LightGBM comparators; the dashed line marks the endpoint-conditioned co-reporting estimate. **(B)** Paired AUPRC differences relative to endpoint-conditioned co-reporting. **(C)** Precision among the 50 highest-ranked rows and top-decile enrichment. **(D)** Brier score and expected calibration error. Error bars and paired differences use clustered resampling by medicinal-product identity. Comparator identity in panels C and D is represented by both the marker shape and color.

The primary supervised benchmark was evaluated under five-fold endpoint- and period-stratified cross-validation (Figure 3; Table 3; Supplementary Table S5). A prevalence-only comparator achieved AUPRC 0.228, near the pooled positive fraction of 0.234 (147/628), whereas conditioning on endpoint and period alone raised the AUPRC to 0.453. The increase reflected substantial variation in the endpoint base rates.

**TABLE 3:** Comparator performance and feature-family analyses.

Question	Comparator or analysis	Results	Interpretation
How strong is a conventional source-signal baseline?	FAERS co-report score with endpoint and period conditioning	AUPRC 0.753; top-decile enrichment 4.07	This is the main reference comparator for model and feature-family analyses
Do transparent multivariable models improve over that baseline?	Elastic-net logistic regression over five FAERS scores plus endpoint and period	AUPRC 0.767; paired difference +0.014 (−0.021 to +0.051)	Comparable performance; the clustered paired-difference interval did not exclude zero *versus* the strongest conditioned source-signal baseline
Do non-linear comparators improve the benchmark?	EBM and LightGBM comparators	EBM AUPRC 0.751; LightGBM AUPRC 0.774	Highest point estimate was obtained from LightGBM, but its paired-difference interval also crossed zero
Do drug class and chemical structure add signal?	Repeated drug-held-out partitions with the boosted-tree comparator	Repeated means: Full FAERS 0.764; +structure 0.796; +structure/drug class 0.800. Single holdout: +0.027 (−0.002 to +0.058)	Modest secondary signal, mainly driven by chemical structure; the single-holdout clustered interval included zero
Does the structure increment extend to unseen chemical scaffolds?	Thirty Bemis–Murcko-scaffold-held-out partitions	Mean structure increment −0.003	No gain when test scaffolds were absent from training
Do target and pathway indicators add further value?	Target and Reactome features after source scores, drug class, and structure	AUPRC 0.801; increment +0.001	No material incremental signal under the tested representation
Does anaphylaxis source ranking enrich future-period Canada reporting-signal-supported pairs?	Permutation test among 1,638 source-exposed anaphylaxis candidates	Co-report mean rank percentile 0.710, $P<10^{-4}$ ; top-decile captured 15/37 manufacturer-screened source-exposed supported pairs	Source-ranking enrichment for anaphylaxis within a small endpoint-specific reporting-signal support set

Among reporting-signal comparators, the raw FAERS co-report score achieved AUPRC 0.613, AUROC 0.788, and top-decile enrichment 2.98. Adding endpoint and period conditioning to the same co-report signal increased AUPRC to 0.753 and top-decile enrichment to 4.07. This endpoint-conditioned co-report comparator became the relevant strong reference point for later models because it produced the single strongest univariate source signal, the same endpoint/period context available to multivariable learners.

The strong endpoint-conditioned co-report comparator left limited room for incremental model gain. Elastic-net logistic regression over the five FAERS source-signal scores and endpoint/period covariates achieved AUPRC 0.767, AUROC 0.879, Brier score 0.105, and top-decile enrichment 3.87. The paired AUPRC difference relative to endpoint-conditioned co-report was +0.014, with a clustered bootstrap interval of −0.021 to +0.051. EBM achieved AUPRC 0.751, with a paired difference of −0.001 and interval of −0.043 to +0.036. LightGBM achieved AUPRC 0.774, with a paired difference of +0.022 and interval of −0.022 to +0.069. No fitted comparator had a clustered paired-difference interval that excluded zero. Endpoint-conditioned co-reporting, therefore, set a demanding reference point for the benchmark.

Endpoint-stratified estimates showed that comparator performance varied with the clinical phenotype (Supplementary Figure S9; Supplementary Table S24). Endpoint-conditioned co-reporting achieved AUPRC 0.899 for anaphylaxis and 0.895 for seizure, compared with 0.620 for acute kidney injury, 0.568 for QT/proarrhythmia, and 0.472 for hepatic injury. Of 15 fitted-model comparisons with endpoint-conditioned co-reporting, only LightGBM for anaphylaxis had a paired interval excluding zero (+0.040; 95% interval +0.002 to +0.097). The remaining endpoint-specific intervals were wide, consistent with the limited number of positives in several strata.

### 3.4 The chemical structure captured information within observed chemical neighborhoods

**FIGURE 4:**
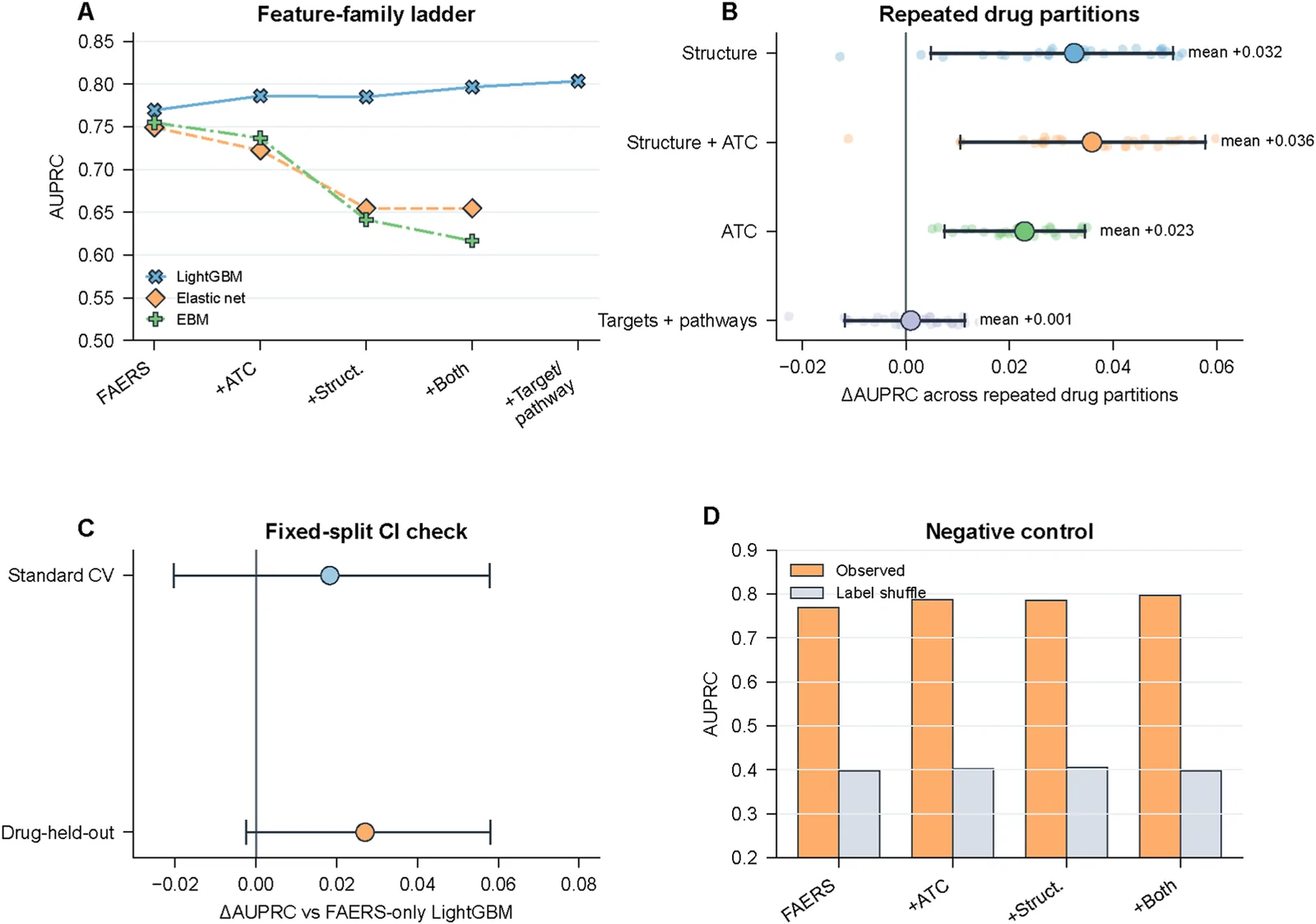
Feature-family augmentation and drug-held-out robustness. **(A)** Feature-family performance in the single drug-held-out split across LightGBM, elastic-net, and EBM comparators; comparator identity is represented by marker shape, line style, and color. **(B)** AUPRC differences across 30 repeated drug-held-out partitions; points show the partition estimates, large markers show the means, and horizontal intervals span the fifth–95th percentiles. **(C)** AUPRC differences for standard cross-validation and a separate drug-held-out split, with 95% medicinal-product-clustered bootstrap intervals. **(D)** Observed and label-shuffled LightGBM performance. Feature-family increments are evaluated relative to the full FAERS source-signal baseline unless otherwise indicated. The +0.015 reference is an exploratory materiality screen rather than a significance threshold; unseen-scaffold results are reported in Supplementary Figure S10.

We next tested whether the drug-class, chemical-structure, target, and pathway features added value over the full FAERS source-signal baseline (Figure 4; Table 3; Supplementary Figure S6). These estimates were obtained from 30 repeated drug-held-out partitions and are distinct from the primary five-fold within-observed-product cross-validation results. The full FAERS source-signal baseline had the mean AUPRC 0.764. Adding chemical structure increased the mean AUPRC to 0.796 (mean difference +0.032), and the combined structure plus drug-class feature set reached 0.800 (mean difference +0.036; Supplementary Table S18).

The gain was mainly structure-driven. Drug class alone reached the mean AUPRC 0.787 (mean difference +0.023), while adding drug class after chemical structure yielded a mean increment of +0.003. Within-partition permutation reduced AUPRC by a mean of 0.050 for ECFP4 features, 0.026 for physicochemical descriptors, and 0.005 for ATC features (Supplementary Figure S10). Under unseen-scaffold partitions, however, the mean structure increment was −0.003, compared with +0.032 under random drug-held-out partitions; the ATC increment beyond structure was −0.002 (Supplementary Table S18). The observed structure increment, therefore, did not extend to test compounds whose chemical scaffolds were absent from training.

Endpoint-conditioned target and Reactome pathway features increased the mean AUPRC from 0.800 to 0.801 (mean +0.001; Supplementary Table S18). Under label shuffling, the structure and drug-class increment collapsed (mean −0.056), and absolute performance reduced to the endpoint- and period-conditioned null range. Elastic-net, EBM, and random-forest comparators did not reproduce the structure/drug-class gain (Supplementary Table S6). In the separate single drug-held-out replication, structure and drug class increased the AUPRC from 0.770 to 0.797, a difference of +0.027 with a medicinal-product-clustered 95% interval of −0.002 to +0.058 (Supplementary Table S18).

The label-efficiency analysis further characterized the operating range of these models under the primary cross-validation design (Supplementary Figure S7; Supplementary Table S13). The endpoint-conditioned co-report comparator achieved the mean AUPRC 0.739 at 25% of training labels and 0.751 at 50%. Elastic-net performance followed a similar pattern. LightGBM was less stable under reduced labels, and chemical-structure augmentation did not exceed the co-report comparator at 25% or 50%. Structure-augmented LightGBM reached the mean AUPRC 0.755 at 75% and 0.795 at 100% of training labels and 0.042 above the endpoint-conditioned co-report comparator at full labels. This 100% estimate was derived from the primary cross-validation design, whereas the 0.796 estimate above was derived from repeated drug-held-out partitions.

### 3.5 Anaphylaxis showed source-ranking enrichment

**FIGURE 5:**
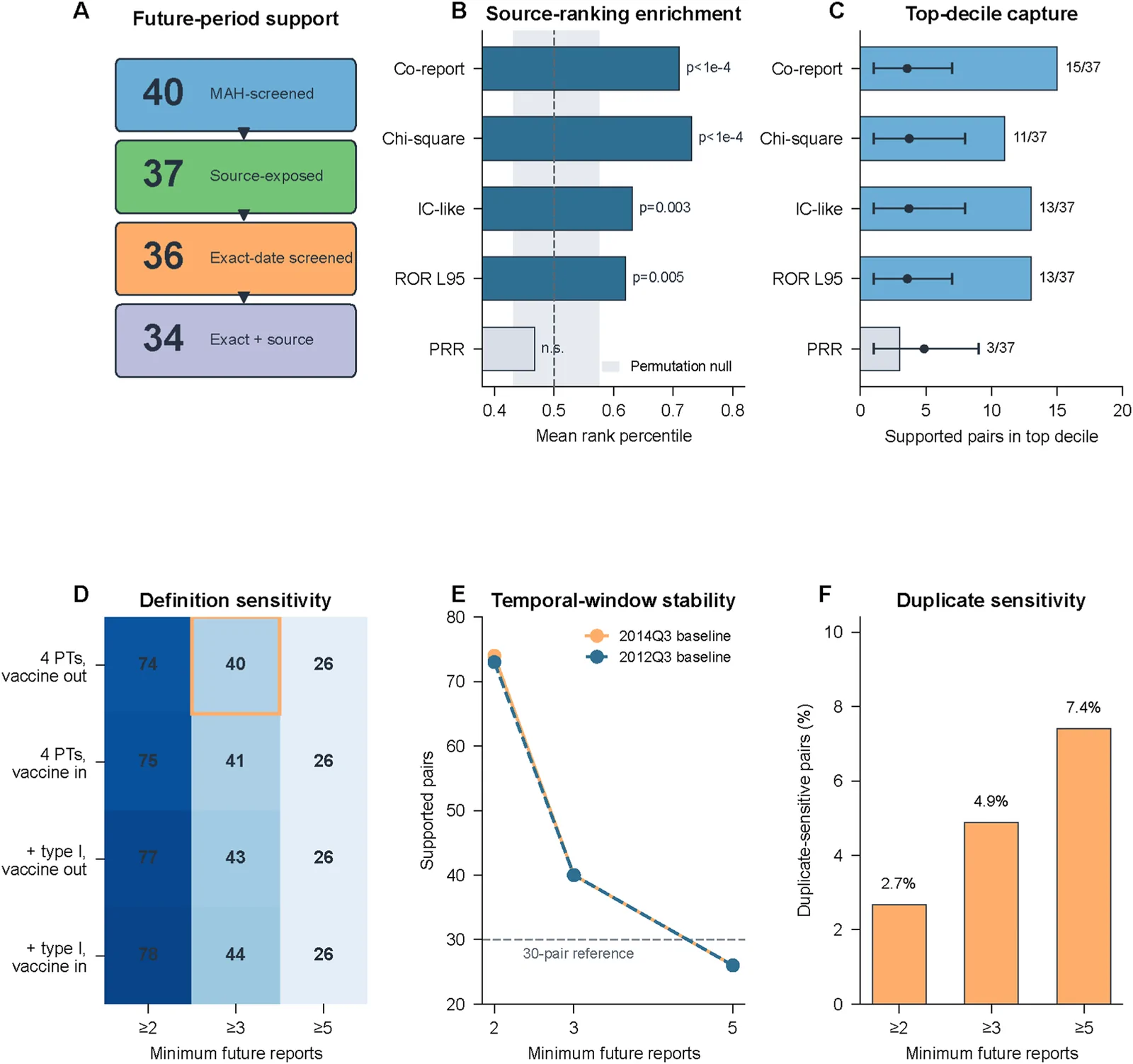
Anaphylaxis future-period support enrichment. **(A)** Manufacturer-screened and exact-date-screened support counts, with and without FAERS source exposure. **(B)** Mean source-score rank percentiles relative to the permutation null. **(C)** Supported pairs captured in the highest-ranked decile. **(D)** Sensitivity to the endpoint definition, vaccine policy, and minimum future-report count. **(E)** Stability across two historical baseline windows; the horizontal line marks the pre-specified 30-pair feasibility reference. **(F)** Fraction of support pairs affected by detectable duplicate screening. No model was fitted in this endpoint-specific analysis.

Anaphylaxis was the only endpoint family with enough manufacturer-screened future support for a separate future-period support analysis (Figure 5). The support ladder contained 40 manufacturer-screened supported pairs, 37 of which also had FAERS source-side exposure. After exact-date non-manufacturer duplicate screening, 36 supported pairs remained, including 34 with FAERS source-side exposure. The analysis used a source-ranking question: among 1,638 source-exposed candidate drug pairs, did FAERS source-side scores rank the future-period Canada Vigilance reporting-signal-supported anaphylaxis pairs above a permutation null?

Four of five FAERS source scores showed significant enrichment. In the manufacturer-screened source-exposed set of 37 pairs, co-report count had a mean rank percentile of 0.710 $\left(P<10^{-4}\right)$ . The permutation null was centered at 0.500. Chi-square had a mean rank percentile of 0.731 $\left(P<10^{-4}\right)$ . The reporting odds ratio lower-confidence bound and the information-component-like score were also above the null, with mean rank percentiles of 0.620 and 0.631. The proportional reporting ratio showed no enrichment (mean rank percentile 0.467). The co-report top-decile captured 15 of 37 supported pairs, compared with 3.6 expected under permutation. The exact-date screened source-exposed view (34 pairs) showed the same qualitative pattern.

## 4 Discussion

This study establishes a cross-system pediatric pharmacovigilance benchmark in which active-ingredient identity, clinical endpoint definitions, and detectable manufacturer cross-filing are resolved before model evaluation. Transparent FAERS source-signal comparators showed strong concordance with manufacturer-screened Canada Vigilance reporting-signal support, and their performance was stable across alternative label definitions. More flexible comparators did not reliably improve on the strongest conditioned source signal. Chemical structure added information when medicinal products, but not chemical scaffolds, were held out, and anaphylaxis supported a focused future-period ranking analysis. These findings define both the utility and the present limits of modeling cross-system pediatric reporting signals from public data.

Pediatric pharmacovigilance is constrained by sparse trial evidence, off-label use, developmental heterogeneity, and incomplete capture of clinically important adverse reactions in routine evidence streams (Black et al., 2015; van der Zanden et al., 2022; Eberl et al., 2024; Ramos et al., 2024). Spontaneous-report systems extend the evidence base for rare, serious, and unexpected events, but they also contain under-reporting, duplicate submissions, selective reporting, missing denominators, and changing product vocabularies (Bailey et al., 2016; Alomar et al., 2020; Giunchi et al., 2023; Khan et al., 2024). These properties directly affect the benchmark labels and estimated model performance. The present study incorporates active-ingredient normalization, clinical MedDRA adjudication, source separation, and manufacturer-key screening into the benchmark definition.

The benchmark addresses a specific question: whether source-side pediatric reporting signals recover drug–endpoint pairs with later manufacturer-screened reporting-signal support in a second system. This concordance target is reproducible from public data and follows recent guidance on transparent case definitions, comparator selection, duplicate handling, and disproportionality analysis (Fusaroli et al., 2024a; Fusaroli et al., 2024b; Jiao et al., 2024). It does not estimate incidence or causal drug risk, which require exposure denominators and designs capable of causal control.

### 4.1 Why strong source-signal baselines matter

Endpoint-conditioned co-reporting provided a demanding reference comparator. Raw co-reporting was already informative, and endpoint and period conditioning brought its performance close to that of the multivariable models. Endpoint prevalence varied substantially, so models and source-signal comparators require the same endpoint context for fair evaluation. The clustered paired-difference intervals showed no reliable improvement from elastic-net, EBM, or LightGBM comparators. Small point-estimate gains should, therefore, be interpreted against the uncertainty of the paired comparison.

This finding was not confined to the primary label specification. Varying report-count minima, signal-family agreement, observability, and borderline-row assignment changed the composition and prevalence of the benchmark but did not yield a consistent advantage for the fitted models. The endpoint-boundary sensitivity definition added only three positive rows, all in historical acute kidney injury, and left positive counts in the other endpoint-period strata unchanged. Endpoint-specific results were more heterogeneous: anaphylaxis and seizure were readily ranked by conditioned co-reporting, while the kidney, hepatic, and QT/proarrhythmia strata had lower AUPRCs and wider intervals. Only the LightGBM comparison in anaphylaxis excluded zero. The pooled result is, therefore, a benchmark-level conclusion rather than evidence that every clinical phenotype has the same operating characteristics.

This pattern is consistent with evidence that classical disproportionality methods remain competitive despite many proposed statistical and machine-learning extensions (Dijkstra et al., 2020; Jiao et al., 2024). The five continuous source features summarized the same FAERS contingency tables and were consequently correlated. The supervised set contained 628 rows and 147 positives distributed across endpoint and period strata, limiting the complexity that could be estimated reliably. Second-system reporting support also reflects utilization, market presence, reporting attention, and coding behavior alongside pharmacology. A strong source-signal baseline can, therefore, capture much of the variation available in this target. More complex models remain useful when they provide a reproducible increment, improved calibration, or clinically interpretable ranking changes; complexity alone is not a sufficient reason for adoption.

### 4.2 The chemical structure adds local, model-specific information

Chemical-structure features contributed a modest increment in boosted-tree models under repeated random drug-held-out partitions. ECFP4 fingerprints accounted for the largest decrease in performance when permuted, followed by physicochemical descriptors; ATC features contributed little beyond structure. The increment disappeared when training and test sets were separated by chemical scaffold. The random-holdout result is, therefore, best understood as interpolation within represented chemical neighborhoods, not as evidence that the model extrapolates to unseen chemical series. The finding remains model-specific. Repeated partitions describe its central tendency across random drug splits, whereas the separate single-holdout clustered interval includes zero and provides the more conservative inferential summary. The increment is, therefore, suggestive rather than conclusive; elastic-net, EBM, and random-forest comparators did not reproduce it.

Target and Reactome pathway indicators added no material information beyond source scores, structure, and drug class under the tested representation. High-dimensional biological annotations may require larger cross-system label sets, exposure or prescribing denominators, route and dose information, and mechanisms aligned more closely with each clinical phenotype. Current pharmacovigilance research increasingly uses knowledge graphs and structured real-world data (Hauben et al., 2024; Farnoush et al., 2024; Tian et al., 2026). The present results show that such representations should be assessed against strong source-signal comparators and under drug-held-out and label-shuffle controls.

The label-efficiency analysis further clarified this operating range. Endpoint-conditioned co-reporting and elastic-net models retained much of their performance under reduced training labels. Boosted-tree models required larger label fractions before chemical-structure information became useful. Pharmacological features, therefore, complemented clinically curated labels in this benchmark; they did not compensate for severe label scarcity.

### 4.3 Future-period support is concentrated in anaphylaxis

Future-period support differed markedly across endpoint families. Anaphylaxis retained sufficient manufacturer-screened support for a separate source-ranking analysis, whereas the four other families remained descriptive in the future stratum. Four FAERS source scores ranked supported anaphylaxis pairs above permutation expectations, with proportional reporting ratio as the exception. The result provides a focused example of future-period cross-system enrichment after manufacturer screening and exact-date sensitivity analysis. It also shows the importance of phenotype definition, vaccine handling, and duplicate screening for pediatric hypersensitivity signals.

A prospective extension should freeze product identities, endpoint definitions, source-feature calculations, model settings, and duplicate-screening rules before a new reporting period accrues. The external system should provide age, report-level seriousness, product and event coding, report dates, and case or manufacturer identifiers that are sufficient to reproduce the target definition. Performance should then be evaluated once on the held-out period with calibration, ranking, and medicinal-product-clustered uncertainty reported under the same analysis plan. This design would preserve the separation between model development and prospective assessment while testing whether source rankings generalize beyond the anaphylaxis-focused demonstration and beyond the regulatory systems used here.

### 4.4 Strengths and limitations

The principal strengths are the separation of source roles, active-ingredient harmonization, clinical endpoint adjudication, detectable cross-filing screening, and common evaluation rules across comparators. Borderline Canada Vigilance evidence remained outside both supervised classes, preserving uncertainty in the label definition. Drug-held-out partitions and label-shuffle controls tested the added feature families under conditions that reduce identity memorization and spurious performance. The released benchmark summaries and source-data tables make these decisions auditable and reusable.

The source data impose several limitations. Spontaneous reports do not provide exposed-person denominators and cannot support incidence or causal risk estimates. Public manufacturer identifiers capture only an observable subset of cross-filed reports. Canada Vigilance manufacturer-key coverage ranged from 52.7% to 72.4% across the benchmark endpoints, leaving residual duplicates or linked reports unresolved. Product normalization also omits the formulation, route, dose, and regimen differences, and some ambiguity persists for incompletely specified products.

An exact numerically usable age was unavailable in 42.0% of FAERS/AERS reports and 23.0% of Canada Vigilance reports. Reports with and without exact age information differed in sex completeness, reporting channel, seriousness, and suspect-ingredient composition, although their most frequent reaction terms overlapped substantially. Requiring convertible report-level age evidence improved the specificity of pediatric eligibility and selected for reports with more complete demographic information. Clinical endpoint adjudication similarly favored specificity, particularly for heterogeneous hepatic-injury and seizure terminology.

Cross-system concordance can reflect shared utilization, market availability, product prominence, reporting attention, and coding practices. Serious-endpoint labels were derived from two North American systems, and JADER contributed descriptive signal-pattern context because its public schema lacks a comparable report-level seriousness field. The primary cross-validation estimates performance within the observed medicinal-product universe and includes historical and future rows in stratified folds. Four endpoint families had insufficient future positive support for powered endpoint-specific temporal evaluation. Independent validation will require additional reporting periods or systems that expose the fields needed to reproduce the same label and duplicate-screening definitions.

The benchmark unit is a drug–endpoint–period row rather than an individual patient. It cannot estimate patient-level prediction performance or equity across demographic groups. Such analyses require individual exposure histories, outcomes, and subgroup labels that are not available in the public spontaneous-report extracts used here. A patient-level extension using linked clinical data should prespecify subgroup analyses by age, sex, clinical complexity, and care setting and should assess subgroup calibration and ranking under patient-level outcomes.

### 4.5 Implications

Pediatric reference sets and resources such as the Osokogu reference set and KidSIDES established important foundations for pediatric signal evaluation and pediatric-specific drug-effect discovery (Osokogu et al., 2015; Giangreco and Tatonetti, 2022). The present benchmark complements those resources by defining a public cross-system reporting-signal target after active-ingredient harmonization, clinical endpoint adjudication, and detectable manufacturer screening. It is intended to compare signal-prioritization methods under common labels, quantify whether new drug features add information beyond conventional reporting statistics, and identify high-ranked pairs for expert assessment. Individual-patient risk estimation and clinical case assessment remain separate tasks.

Future releases should retain versioned product mappings and endpoint assignments, document changes in source coverage, and route new or altered MedDRA terms through the same clinical review used for the initial definitions. This preserves comparability across benchmark versions while allowing new pediatric outcomes and reporting periods to be added.

In summary, this study establishes a manufacturer-aware, clinically adjudicated benchmark for pediatric pharmacovigilance signal concordance. Transparent endpoint-conditioned source signals set a strong reference, chemical structure added a modest secondary increment, and anaphylaxis provided a focused future-period enrichment example. The resulting public-data resource supports reproducible comparison of pediatric drug-safety signal methods.

## Data Availability

The datasets presented in this study can be found in online repositories. The names of the repository/repositories and accession number(s) can be found below: the benchmark source-data tables, analysis manifests, and figure-source tables generated for this study are available in Zenodo (doi:10.5281/zenodo.21634673), and the analysis code is available at https://github.com/BowenShiGDPU/Pediatric-pharmacovigilance-signal-concordance-benchmark. The underlying public pharmacovigilance extracts are available from their original providers: FAERS/AERS from the U.S. Food and Drug Administration, Canada Vigilance from Health Canada, and JADER from the Pharmaceuticals and Medical Devices Agency.
