# Supplementary material for: A manufacturer-aware cross-system benchmark for pediatric pharmacovigilance signal concordance
Source: Front Pharmacol. 2026 Sep 7;17:1900271. doi: 10.3389/fphar.2026.1900271 (PMC13590764; doi:10.3389/fphar.2026.1900271)
Supplement: Supplementary file 1 [file DataSheet1.pdf]

Supplementary Material

This file provides supporting tables and figures for endpoint adjudication, source comparability, benchmark construction, label-definition sensitivity, endpoint-specific uncertainty, feature-family analyses, label efficiency, and anaphylaxis enrichment. Counts are public spontaneous-report counts or derived benchmark summaries rather than incidence rates.

Supplementary Table S1. Endpoint preferred-term adjudication agreement and final consensus counts. Candidate MedDRA preferred terms were independently assigned to primary endpoint, sensitivity-only, or excluded categories by two adjudicators with training in pharmacovigilance, pediatric medication safety, and clinical terminology review. Disagreements were resolved by a third adjudicator using MedDRA hierarchy, endpoint definitions, and pediatric clinical plausibility.

Endpoint family	Candidate PTs	Initial agreement	Cohen's κ (95% CI)	Third-adjudicated PTs	Final primary / sensitivity / excluded PTs
Acute kidney injury	18	83.3%	0.74 (0.42–1.00)	3	3 / 9 / 6
Anaphylaxis	6	100.0%	1.00 (1.00–1.00)	0	4 / 1 / 1
Hepatic injury	157	92.4%	0.83 (0.73–0.91)	12	25 / 22 / 110
QT/proarrhythmia	13	100.0%	1.00 (1.00–1.00)	0	6 / 3 / 4
Seizure	41	95.1%	0.91 (0.78–1.00)	2	25 / 13 / 3

Supplementary Table S2. Public-source preprocessing definitions. The three public reporting systems were assigned distinct roles that preserve their public-schema differences.

Source	Study role	Main public fields used	Scope boundary
FAERS/AERS	Source-side pediatric reporting signals	Report demographics, suspected medicinal products, reaction preferred terms, seriousness/outcome fields, reporter information, and manufacturer report identifiers	Ages in years, months, weeks, days, or hours were converted to years with standard unit factors; decade-coded ages were multiplied by 10. Pediatric reports required converted age <18 years; missing, inconsistent, or non-convertible ages were excluded from the pediatric serious-label universe. Seriousness used public seriousness/outcome fields.
Canada Vigilance	Manufacturer-screened reporting-signal comparator	Report-level seriousness, medicinal-product records, reaction terms, demographics, and manufacturer authorization holder numbers	Pediatric serious reports required convertible age <18 years and the public serious-report indicator. Manufacturer keys were upper-cased, trimmed, reduced to alphanumeric characters, and matched exactly; sender prefixes, fuzzy company matching, and inferred manufacturer links were not used. Public fields identify detectable rather than complete de-duplication.
JADER	Descriptive third-country landscape	MedDRA/J reaction terms, categorical age/sex fields, and public product records	Used for signal-pattern context because the public schema lacks a comparable report-level seriousness field and manufacturer-control key; categorical age bands were kept in their native public form
Reference vocabularies	Harmonization resources	RxNorm, DrugCentral, ChEMBL, MedDRA, ATC, Reactome, and chemical structure descriptors	Used for product identity, endpoint definitions, drug class, structure, target, and pathway features; controlled vocabularies were summarized without redistributing restricted files

Supplementary Table S3. Drug–event signal and label definitions. Signals were computed separately within source, endpoint, and period strata before constructing supervised benchmark rows.

Quantity	Operational definition	Use in benchmark
Drug–event contingency table	For each medicinal product, endpoint family, and period: a = reports with the product and endpoint; b = product reports without the endpoint; c = endpoint reports without the product; d = reports with neither.	Basis for source-side FAERS features and Canada target signals
Co-report count	The drug–endpoint cell count a.	Used as a source-side score, a minimum observability check, and an anaphylaxis enrichment ranking score
Reporting odds ratio lower bound	Lower 95% confidence bound of the reporting odds ratio derived from the same 2×2 table with 0.5 added to each cell.	One signal family; signal-positive when $a \geq 3$ and the lower bound was greater than 1
Proportional reporting ratio	Ratio of the endpoint fraction among product reports to the endpoint fraction among non-product reports, using the same 0.5 cell correction.	One signal family and a standalone ranking comparator; signal-positive when $a \geq 3$, PRR was at least 2, and chi-square was greater than 4
Information-component-like score	Log-ratio of observed to expected drug–endpoint co-reporting under independence, with 0.5 added to observed and expected counts.	One signal family and a standalone ranking comparator; signal-positive when $a \geq 3$ and the score was greater than 0
Two-of-three source signal	A binary source-side signal requiring $a \geq 3$ and agreement of at least two families: reporting-odds-ratio lower bound > 1; PRR ≥ 2 with chi-square > 4; and information-component-like score > 0.	Required for a row to enter the primary source-exposed benchmark universe
Primary positive label	FAERS source exposure plus at least three Canada drug–endpoint reports after detectable manufacturer screening and a positive Canada two-of-three signal.	Manufacturer-screened Canada reporting-signal positive class for supervised benchmark metrics
Canada co-report-absent label	FAERS source exposure plus zero Canada drug–endpoint reports after detectable manufacturer screening.	Canada co-report-absent comparator class for supervised benchmark metrics; interpreted as absence of public Canada co-report evidence
Borderline target evidence	One or two Canada drug–endpoint reports after detectable manufacturer screening, or target evidence below the primary positive rule.	Withheld from primary supervised metrics and retained for sensitivity/accounting

Supplementary Table S4. Supervised benchmark row accounting by endpoint family. Rows are medicinal-product–endpoint–period units after active-ingredient normalization, endpoint consensus adjudication, manufacturer screening, and reservation of the anaphylaxis support-enrichment set.

Endpoint family	Positive rows	Canada co-report-absent rows	Withheld rows	Interpretation note
Acute kidney injury	32	122	42	Narrow primary definition; endpoint-specific summaries interpreted with caution because term-adjudication agreement was lowest among retained endpoint families
Anaphylaxis	34	24	32	Supervised rows are disjoint from the separate anaphylaxis support-enrichment set
Hepatic injury	17	156	46	Clinically specific hepatic injury terms retained; laboratory-only and infectious/perinatal contaminants excluded from primary labels
QT/proarrhythmia	22	124	25	QT prolongation and ventricular arrhythmia terms retained; nonspecific arrest terms outside primary labels
Seizure	42	55	30	Organic seizure terms retained; febrile, metabolic, psychogenic, indication, and infectious-syndrome terms outside primary labels
Total	147	481	175	Primary supervised metrics use 628 positive or Canada co-report-absent rows; withheld rows are retained outside the primary comparator class, and the anaphylaxis enrichment set is analyzed separately

Supplementary Table S5. Comparator, feature, and uncertainty specification. All model inputs were source-side or pre-outcome covariates; Canada-derived target columns and post-outcome adverse-event knowledge were excluded from predictors.

Analysis component	Included inputs or models	Evaluation role
Reference source-signal comparators	Prevalence-only, endpoint/period prevalence, raw FAERS single-score rankers, and endpoint/period-conditioned single-score logistic comparators	Establish the strongest transparent pharmacovigilance reference baselines
Interpretable models	Elastic-net logistic regression over five FAERS source scores plus endpoint and period covariates; explainable boosting machine	Test whether transparent multivariable learners materially improve over the strongest conditioned source-signal comparator
Gradient-boosted tree comparator	LightGBM with hyperparameters selected inside training folds only	Nonlinear reference learner for comparator evaluation
Model tuning and specificity check	Elastic-net mixtures and regularization strengths and small EBM/LightGBM grids were selected within training data; the random-forest specificity comparator used 300 trees, maximum depth 6, minimum leaf size 8, and balanced class subsampling	Documents model-selection constraints and the model-family specificity comparator
Core source features	FAERS co-report count, reporting odds ratio lower confidence bound, proportional reporting ratio, chi-square statistic, information-component-like score, endpoint family, and period	Primary supervised benchmark features
Fold-level preprocessing	Training-fold median imputation for continuous predictors; training-fold most-frequent-category imputation followed by one-hot encoding for categorical predictors; scaling fitted in the training fold	Prevents test-fold information from entering missing-value handling, encoding, or scaling
Feature-family augmentation	First-level ATC indicators; radius-2 Morgan/ECFP4 fingerprints with 1,024 raw bits and supervised-drug minimum-support filtering; seven physicochemical descriptors; target indicators; and Reactome pathway indicators, added incrementally to the full FAERS baseline	Secondary analysis of whether pharmacological context adds value beyond source reporting signals
Uncertainty and robustness	Medicinal-product-clustered bootstrap, label-shuffle controls, and repeated drug-held-out partitions for feature-family analyses	Prevent row-level dependence, label leakage, and drug-identity memorization from driving claims

Supplementary Table S6. Minimal public release contents. The release is designed to reproduce manuscript summaries without redistributing controlled vocabularies or raw regulatory extracts.

Release component	Contents	Purpose
Checking code	Lightweight scripts for checking table schema, row counts, checksums, and figure-source-data consistency	Allows readers to check released summaries without accessing private working files
Benchmark summary tables	Endpoint label accounting, endpoint adjudication agreement, model metrics, feature-family summaries, and anaphylaxis enrichment outputs	Supports the main tables and Results claims
Source-data tables	Panel-level source tables for all main figures, supplementary figures, and supplementary tables	Supports graphical and tabular reproduction and journal source-data requirements
Source-data documentation	Data dictionary, source-data manifest, release README, and repository/DOI metadata	Describes how public outputs map to the manuscript while avoiding redistribution of licensed vocabularies or raw case-level extracts
Excluded materials	Raw FAERS, Canada Vigilance, JADER extracts; MedDRA and ATC controlled-vocabulary files; private intermediate files; and patient-level narratives	Limits redistribution to derived, documented research outputs

Supplementary Table S7. Primary endpoint preferred-term lists. These MedDRA preferred terms were used to construct the supervised endpoint labels. Complete candidate-level assignments, including sensitivity-only and excluded terms, are reported in Supplementary Table S14 and the released source-data table. Selected non-primary examples clarify the principal adjudication boundaries.

Endpoint family	Primary preferred terms
Acute kidney injury	Acute kidney injury; RENAL FAILURE ACUTE; RENAL TUBULAR NECROSIS
Anaphylaxis	ANAPHYLACTIC REACTION; ANAPHYLACTIC SHOCK; ANAPHYLACTOID REACTION; ANAPHYLACTOID SHOCK
Hepatic injury	ACUTE HEPATIC FAILURE; ALLERGIC HEPATITIS; CHOLESTASIS; CHOLESTATIC LIVER INJURY; CYTOLYTIC HEPATITIS; DRUG-INDUCED LIVER INJURY; Eosinophilic hepatitis; Hepatic cytolysis; HEPATIC FAILURE; HEPATIC NECROSIS; HEPATITIS ACUTE; HEPATITIS CHOLESTATIC; HEPATITIS FULMINANT; HEPATITIS TOXIC; HEPATOTOXICITY; Immune-mediated cholestasis; Immune-mediated hepatitis; JAUNDICE; JAUNDICE CHOLESTATIC; JAUNDICE HEPATOCELLULAR; LIVER INJURY; MIXED HEPATOCELLULAR-CHOLESTATIC INJURY; MIXED LIVER INJURY; SUBACUTE HEPATIC FAILURE; Suspected drug-induced liver injury
QT/proarrhythmia	ELECTROCARDIOGRAM QT CORRECTED INTERVAL PROLONGED; ELECTROCARDIOGRAM QT PROLONGED; TORSADE DE POINTES; VENTRICULAR ARRHYTHMIA; VENTRICULAR FIBRILLATION; VENTRICULAR TACHYCARDIA
Seizure	Atypical absence seizure; AUTONOMIC SEIZURE; CLONIC CONVULSION; COMPLEX PARTIAL SEIZURES; CONVULSION; CONVULSION IN CHILDHOOD; CONVULSION NEONATAL; Faciobrachial dystonic seizure; Gelastic seizure; Generalised onset non-motor seizure; Generalised tonic-clonic seizure; GRAND MAL CONVULSION; Hemiclonic seizure; Neonatal epileptic seizure; Neonatal seizure; New onset refractory status epilepticus; PARTIAL SEIZURES; PARTIAL SEIZURES WITH SECONDARY GENERALISATION; Photosensitive seizure; Seizure; SEIZURE CLUSTER; SIMPLE PARTIAL SEIZURES; STATUS EPILEPTICUS; Tardive seizure; TONIC CONVULSION

Notes: For QT/proarrhythmia, the primary list retained diagnostic QT prolongation and ventricular arrhythmia terms and excluded congenital long-QT terminology and nonspecific arrest/death terms. For acute kidney injury, the primary list retained acute renal-injury terms and excluded laboratory-only, chronic, fetal/perinatal, neonatal, and viral renal terms.

Endpoint family	Selected non-primary examples	Consensus rationale
Anaphylaxis	Type I hypersensitivity	Sensitivity-only because it belongs to a broader allergic-condition hierarchy rather than the narrow anaphylactic/anaphylactoid response set.
Acute kidney injury	Blood creatinine increased; glomerular filtration rate decreased; renal failure; renal impairment; sepsis-associated acute kidney injury; tubulointerstitial nephritis; renal failure chronic; polyomavirus interstitial nephritis; foetal renal impairment	Laboratory-only, broader renal impairment/failure, chronic, viral, and fetal/perinatal terms were kept outside the primary AKI label.
Hepatic injury	Alanine or aspartate aminotransferase increased; bilirubin increased; viral, alcoholic, pregnancy-related, radiation-related, or traumatic hepatic terms	Laboratory-only findings and non-drug or context-specific mimics were kept outside primary hepatic injury.
QT/proarrhythmia	Cardiac arrest; sudden cardiac death; long QT syndrome; long QT syndrome congenital	Nonspecific arrest/death terms and long-QT disease terminology were kept outside the primary acquired proarrhythmia scope.
Seizure	Febrile convulsion; metabolic seizure terms; psychogenic seizure; seizure prophylaxis	Fever-associated, metabolic, psychogenic, indication, and infectious-syndrome terms were kept outside the primary organic seizure label.

Supplementary Table S8. Endpoint-level manufacturer cross-filing and duplicate-screening analysis. Canada pediatric serious reports are endpoint-specific report counts. Manufacturer-key coverage is the fraction of Canada endpoint reports with a public manufacturer authorization holder number; detectable cross-filing is the fraction with a manufacturer key also observed in FAERS. Manufacturer keys were upper-cased and reduced to alphanumeric characters before exact matching. The support columns summarize threshold-3 future support before and after manufacturer-key screening.

Endpoint	Canada reports	Key coverage (%)	Detectable cross-filing (%)	Naive support	Manufacturer-screened support	Pairs removed by manufacturer-key screening
Acute kidney injury	263	55.5	11.0	2	0	2
Anaphylaxis	744	52.7	5.5	41	40	1
Hepatic injury	386	62.4	8.5	6	3	3
QT/proarrhythmia	185	72.4	14.1	8	7	1
Seizure	1,405	70.1	7.3	9	7	2

Supplementary Table S9. Medicinal-product identity harmonization quality control. Counts summarize candidate identities, analysis eligibility, structure-key availability, and resolved identity conflicts after active-ingredient normalization. Examples show the identity rules applied before benchmark construction.

Quality-control item	Value	Details
Candidate normalized medicinal-product identities	3,681	2,087 primary-axis eligible; 3,681 sensitivity-axis eligible; 2,081 with full InChIKey.
Harmonized medicinal-product identities	3,680	2,086 primary-axis eligible after conflict resolution; 2,081 with full InChIKey.
High-frequency provenance rows	500	Identity provenance was checked for the highest-frequency rows; 424 had a full InChIKey.
Resolved identity conflicts	6	Six identity conflicts were resolved before benchmark construction.
Salt/base and hydrate handling	Rule-based parent normalization	Base, salt, and hydrate variants were harmonized before structural key assignment when they represented the same active moiety.
Combination-product handling	Component-level representation	Combination products were represented by resolved active ingredients when all components were identifiable; unresolved combinations were not used for primary structure-dependent comparisons.
Conflict protection	Keep separate unless identity was confirmed	Prodrug/active-metabolite, racemate/enantiomer, and ambiguous family-name cases could not merge solely through synonym or shared-key edges.

Supplementary Table S10. Anaphylaxis future-period support and source-ranking analysis. The first rows define the support ladder; subsequent rows summarize permutation-based rank enrichment among source-exposed candidate pairs. The ranking analysis used fixed source scores.

Analysis item	Value	Interpretation
Manufacturer-screened supported pairs	40	Anaphylaxis future-period support profile
Exact-date screened supported pairs	36	Anaphylaxis future-period support profile
Manufacturer-screened source-exposed supported pairs	37	Anaphylaxis future-period support profile
Exact-date screened source-exposed supported pairs	34	Anaphylaxis future-period support profile
Future reports after manufacturer screening	225	Anaphylaxis future-period support profile
Future reports after exact-date screening	211	Anaphylaxis future-period support profile
FAERS co-report count	Mean rank percentile 0.710; empirical $P < 10^{-4}$	Manufacturer-screened source-exposed support set; 10,000 permutations.
FAERS reporting odds ratio lower confidence bound	Mean rank percentile 0.620; empirical $P = 0.005$	Manufacturer-screened source-exposed support set; 10,000 permutations.
FAERS proportional reporting ratio	Mean rank percentile 0.467; empirical $P = 0.759$	Manufacturer-screened source-exposed support set; 10,000 permutations.
FAERS chi-square statistic	Mean rank percentile 0.731; empirical $P < 10^{-4}$	Manufacturer-screened source-exposed support set; 10,000 permutations.
FAERS information-component-like score	Mean rank percentile 0.631; empirical $P = 0.003$	Manufacturer-screened source-exposed support set; 10,000 permutations.
Co-report top-decile capture	15/37 supported pairs	Top 10% of FAERS co-report ranking.

Supplementary Table S11. Worked examples of benchmark decision rules. The examples illustrate label construction and do not estimate causal or comparative drug risk.

Example row or row group	Period	Source-side signal shown in benchmark	Canada target label	Interpretation boundary
Ibuprofen–acute kidney injury	Future period	358 FAERS co-reports; ROR lower confidence bound 5.88	Positive	Illustrates a source-exposed positive benchmark row after active-ingredient harmonization and manufacturer-screened Canada evidence.
Ceftriaxone–anaphylaxis	Future period	30 FAERS co-reports; ROR lower confidence bound 2.24	Positive	Illustrates an anaphylaxis row contributing to the future-period support profile under the narrow anaphylaxis endpoint definition.
Moxifloxacin–QT/proarrhythmia	Future period	57 FAERS co-reports; ROR lower confidence bound 43.94	Positive	Illustrates that diagnostic QT prolongation and ventricular arrhythmia terms were evaluated under a narrow acquired proarrhythmia endpoint.
Levetiracetam–seizure	Future period	369 FAERS co-reports; ROR lower confidence bound 6.32	Positive	Illustrates a seizure endpoint row after febrile, metabolic, psychogenic, prophylaxis, and infectious-syndrome mimics were kept outside the primary label.
Acetaminophen across endpoint families	Future period	266 FAERS co-reports for acute kidney injury; 692 for hepatic injury	Acute kidney injury negative; hepatic injury positive	Illustrates endpoint-specific labeling for the same medicinal product rather than assigning a single product-level safety label.

Anaphylaxis historical window	Naive support	Manufacturer-screened support	Pairs removed
2012Q3–2020Q4	41	40	1
2014Q3–2020Q4	41	40	1

Supplementary Table S12. Clinical and pharmacological context for endpoint-family selection. Endpoint families were selected for serious pediatric relevance, pharmacological plausibility, and feasibility of narrow MedDRA preferred-term definitions in public spontaneous reports. The table summarizes the clinical scope used for benchmark construction and the main mimic classes kept outside the primary labels.

Endpoint family	Clinical and pharmacological rationale	Primary endpoint scope	Main boundary exclusions
Acute kidney injury	Drug-induced kidney injury is a serious pediatric safety concern because renal clearance, volume status, nephrotoxic co-exposures, critical illness, and developmental renal physiology can modify susceptibility.	Acute kidney injury, acute renal failure, and renal tubular necrosis.	Laboratory-only renal function changes, chronic renal failure, fetal/perinatal renal impairment, viral nephritis, neonatal renal impairment, and broader renal disease terms.
Anaphylaxis	Anaphylaxis is an acute, potentially life-threatening immune-mediated reaction that is clinically salient in pediatric pharmacovigilance and commonly represented by narrow MedDRA preferred terms.	Anaphylactic or anaphylactoid reaction and shock terms.	Broader hypersensitivity terms, vasculitis, nonspecific allergy terms, and vaccine-focused analyses outside the primary drug benchmark.
Hepatic injury	Drug-induced liver injury is a core post-marketing safety phenotype, but pediatric reports can be confounded by infection, perinatal jaundice, laboratory findings, and underlying liver disease.	Clinically coded hepatic failure, cholestatic/hepatocellular injury, toxic or drug-induced hepatitis, hepatic necrosis, hepatotoxicity, and jaundice-related injury phenotypes.	Laboratory-only liver tests, viral hepatitis, pregnancy/perinatal terms, alcoholic/radiation/traumatic hepatic injury, and broad non-specific mimics.
QT/proarrhythmia	Drug-induced QT prolongation and ventricular proarrhythmia are mechanistically linked to electrophysiological liability and are clinically important for pediatric medication safety.	ECG QT prolongation, torsade de pointes, ventricular tachycardia, ventricular fibrillation, and ventricular arrhythmia.	Long-QT disease terminology, nonspecific cardiac arrest, sudden death, fetal cardiac arrest, and broad non-arrhythmic cardiac outcomes.
Seizure	Drug-associated seizures may reflect direct central nervous system effects, toxicity, withdrawal, or metabolic susceptibility, but pediatric seizure terminology contains many fever-, infection-, and indication-related mimics.	Organic seizure, convulsion, status epilepticus, focal/generalized seizure, neonatal seizure, and related clinically coded seizure phenotypes.	Febrile, metabolic, psychogenic, anoxic, post-stroke, prophylaxis, and infectious-syndrome seizure terms outside the primary endpoint.

Clinical framework sources. Anaphylaxis boundaries were informed by the World Allergy Organization 2020 guidance (doi:10.1016/j.waojou.2020.100472); acute kidney injury by the KDIGO acute kidney injury guideline and a pediatric AKI review (doi:10.23876/j.krcp.20.074); hepatic injury by the AASLD drug-induced liver injury practice guidance (doi:10.1002/hep.32689); QT/proarrhythmia by the 2022 ICH E14/S7B questions and answers and clinical QT-management guidance (doi:10.1136/postgradmedj-2020-138661); and seizure by the 2025 International League Against Epilepsy classification (doi:10.1111/epi.18338). These sources defined clinical phenotype boundaries; final MedDRA preferred-term membership was established independently by two adjudicators, with disagreements resolved by a third adjudicator, as reported in Supplementary Tables S1 and S14.

Supplementary Table S13. Label-efficiency analysis. Values are mean AUPRC across 30 repeated training-label subsamples for fractions below 100%; the 100% rows reproduce the full-label benchmark values. Model definitions and test folds match the main benchmark.

Comparator	25% labels	50% labels	75% labels	100% labels	Interpretation
Prevalence only	0.228	0.229	0.229	0.228	Pooled positive-fraction floor.
Co-report + endpoint/period	0.739	0.751	0.754	0.753	Label-efficient transparent source-signal comparator.
Elastic net	0.740	0.761	0.762	0.767	Closely tracked the conditioned co-report comparator.
LightGBM	0.664	0.726	0.747	0.774	Less stable than transparent comparators under reduced labels.
LightGBM + chemical structure	0.640	0.728	0.755	0.795	Structure features added value mainly at higher label fractions.

Supplementary Table S14. Complete candidate-level MedDRA preferred-term adjudication record. The table reports the two independent assignments, third-adjudicator resolution where required, the boundary-sensitivity role summarized in Supplementary Table S16, and the primary benchmark role.

Endpoint	Candidate preferred term	MedDRA system organ class	Adjudicator 1	Adjudicator 2	Third adjudicator	Boundary-sensitivity role	Primary role and reason
Anaphylaxis	ANAPHYLACTIC REACTION	Immune system disorders	Primary	Primary	Not required	Primary	Primary; core endpoint
Anaphylaxis	ANAPHYLACTIC SHOCK	Immune system disorders	Primary	Primary	Not required	Primary	Primary; core endpoint
Anaphylaxis	ANAPHYLACTOID REACTION	Immune system disorders	Primary	Primary	Not required	Primary	Primary; core endpoint
Anaphylaxis	ANAPHYLACTOID SHOCK	Immune system disorders	Primary	Primary	Not required	Primary	Primary; core endpoint
Anaphylaxis	Hypersensitivity vasculitis	Skin and subcutaneous tissue disorders	Excluded	Excluded	Not required	Sensitivity	Excluded; wrong endpoint or unrelated
Anaphylaxis	TYPE I HYPERSENSITIVITY	Immune system disorders	Sensitivity	Sensitivity	Not required	Sensitivity	Sensitivity; nonspecific broad
Acute kidney injury	Acute kidney injury	Renal and urinary disorders	Primary	Primary	Not required	Primary	Primary; core endpoint
Acute kidney injury	Adenovirus interstitial nephritis	not available in Canada extract	Excluded	Excluded	Not required	Excluded	Excluded; infectious mimic
Acute kidney injury	BLOOD CREATININE INCREASED	Investigations	Sensitivity	Sensitivity	Not required	Sensitivity	Sensitivity; lab only
Acute kidney injury	CREATININE RENAL CLEARANCE DECREASED	Investigations	Sensitivity	Sensitivity	Not required	Sensitivity	Sensitivity; lab only
Acute kidney injury	Foetal renal impairment		Excluded	Excluded	Not required	Excluded	Excluded; pregnancy fetal perinatal
Acute kidney injury	GLOMERULAR FILTRATION RATE DECREASED	Investigations	Sensitivity	Sensitivity	Not required	Sensitivity	Sensitivity; lab only
Acute kidney injury	POLYOMAVIRUS INTERSTITIAL NEPHRITIS	not available in Canada extract	Excluded	Excluded	Not required	Excluded	Excluded; infectious mimic
Acute kidney injury	POSTOPERATIVE RENAL FAILURE		Sensitivity	Sensitivity	Not required	Sensitivity	Sensitivity; metabolic or secondary cause
Acute kidney injury	RENAL FAILURE	Renal and urinary disorders	Sensitivity	Sensitivity	Not required	Sensitivity	Sensitivity; nonspecific broad
Acute kidney injury	RENAL FAILURE ACUTE	not available in Canada extract	Primary	Primary	Not required	Primary	Primary; core endpoint
Acute kidney injury	RENAL FAILURE CHRONIC	not available in Canada extract	Excluded	Excluded	Not required	Excluded	Excluded; chronic or underlying disease
Acute kidney injury	RENAL FAILURE NEONATAL	Renal and urinary disorders	Excluded	Excluded	Not required	Sensitivity	Excluded; pregnancy fetal perinatal
Acute kidney injury	RENAL IMPAIRMENT	Renal and urinary disorders	Sensitivity	Sensitivity	Not required	Sensitivity	Sensitivity; nonspecific broad
Acute kidney injury	RENAL IMPAIRMENT NEONATAL	Renal and urinary disorders	Excluded	Excluded	Not required	Sensitivity	Excluded; pregnancy fetal perinatal
Acute kidney injury	RENAL TUBULAR NECROSIS	Renal and urinary disorders	Primary	Primary	Not required	Primary	Primary; core endpoint
Acute kidney injury	Sepsis associated acute kidney injury		Sensitivity	Excluded	Sensitivity	Primary	Sensitivity; infectious or secondary context
Acute kidney injury	TUBULOINTERSTITIAL NEPHRITIS	Renal and urinary disorders	Sensitivity	Primary	Sensitivity	Primary	Sensitivity; nonspecific broad
Acute kidney injury	TUBULOINTERSTITIAL NEPHRITIS AND UVEITIS SYNDROME	Renal and urinary disorders	Excluded	Sensitivity	Sensitivity	Primary	Sensitivity; syndrome or secondary context
Hepatic injury	ACUTE HEPATIC FAILURE	Hepatobiliary disorders	Primary	Primary	Not required	Primary	Primary; core endpoint
Hepatic injury	Acute hepatitis B		Excluded	Excluded	Not required	Excluded	Excluded; infectious mimic
Hepatic injury	Acute hepatitis C		Excluded	Excluded	Not required	Excluded	Excluded; infectious mimic
Hepatic injury	Acute on chronic liver failure		Primary	Sensitivity	Primary	Primary	Sensitivity; chronic or underlying disease
Hepatic injury	ADENOVIRAL HEPATITIS	Infections and infestations	Excluded	Excluded	Not required	Excluded	Excluded; infectious mimic

Endpoint	Candidate preferred term	MedDRA system organ class	Adjudicator 1	Adjudicator 2	Third adjudicator	Boundary-sensitivity role	Primary role and reason
Hepatic injury	ALANINE AMINOTRANSFERASE INCREASED	Investigations	Sensitivity	Sensitivity	Not required	Sensitivity	Sensitivity; lab only
Hepatic injury	ALLERGIC HEPATITIS		Primary	Primary	Not required	Primary	Primary; core endpoint
Hepatic injury	Alloimmune hepatitis		Excluded	Excluded	Not required	Primary	Excluded; chronic or underlying disease
Hepatic injury	ASPARTATE AMINOTRANSFERASE INCREASED	Investigations	Sensitivity	Sensitivity	Not required	Sensitivity	Sensitivity; lab only
Hepatic injury	ASYMPTOMATIC VIRAL HEPATITIS	not available in Canada extract	Excluded	Excluded	Not required	Excluded	Excluded; infectious mimic
Hepatic injury	AUTOIMMUNE HEPATITIS	Hepatobiliary disorders	Primary	Excluded	Primary	Primary	Sensitivity; disease or immune mimic
Hepatic injury	Benign recurrent intrahepatic cholestasis	not available in Canada extract	Excluded	Excluded	Not required	Excluded	Excluded; chronic or underlying disease; congenital or developmental
Hepatic injury	BILIRUBIN CONJUGATED INCREASED	Investigations	Sensitivity	Sensitivity	Not required	Sensitivity	Sensitivity; lab only
Hepatic injury	BLOOD BILIRUBIN INCREASED	Investigations	Sensitivity	Sensitivity	Not required	Sensitivity	Sensitivity; lab only
Hepatic injury	BLOOD BILIRUBIN UNCONJUGATED INCREASED	Investigations	Sensitivity	Sensitivity	Not required	Sensitivity	Sensitivity; lab only
Hepatic injury	CHOLESTASIS	Hepatobiliary disorders	Primary	Primary	Not required	Primary	Primary; core endpoint
Hepatic injury	CHOLESTASIS OF PREGNANCY		Excluded	Excluded	Not required	Excluded	Excluded; pregnancy fetal perinatal
Hepatic injury	CHOLESTATIC LIVER INJURY	Hepatobiliary disorders	Primary	Primary	Not required	Primary	Primary; core endpoint
Hepatic injury	CHOLESTATIC PRURITUS	Skin and subcutaneous tissue disorders	Sensitivity	Sensitivity	Not required	Sensitivity	Sensitivity; nonspecific broad
Hepatic injury	CHRONIC HEPATIC FAILURE		Primary	Excluded	Sensitivity	Sensitivity	Sensitivity; chronic or underlying disease
Hepatic injury	CHRONIC HEPATITIS	Hepatobiliary disorders	Primary	Excluded	Sensitivity	Sensitivity	Sensitivity; chronic or underlying disease
Hepatic injury	Chronic hepatitis B	Infections and infestations	Excluded	Excluded	Not required	Excluded	Excluded; infectious mimic
Hepatic injury	Chronic hepatitis C		Excluded	Excluded	Not required	Excluded	Excluded; infectious mimic
Hepatic injury	Congenital hepatitis B infection	not available in Canada extract	Excluded	Excluded	Not required	Excluded	Excluded; infectious mimic
Hepatic injury	CYTOLYTIC HEPATITIS	not available in Canada extract	Primary	Primary	Not required	Primary	Primary; core endpoint
Hepatic injury	CYTOMEGALOVIRUS HEPATITIS	Infections and infestations	Excluded	Excluded	Not required	Excluded	Excluded; infectious mimic
Hepatic injury	DRUG-INDUCED LIVER INJURY	Hepatobiliary disorders	Primary	Primary	Not required	Primary	Primary; core endpoint
Hepatic injury	Eosinophilic hepatitis	not available in Canada extract	Primary	Primary	Not required	Primary	Primary; core endpoint
Hepatic injury	Hepatic cytolysis	Hepatobiliary disorders	Primary	Primary	Not required	Primary	Primary; core endpoint
Hepatic injury	HEPATIC FAILURE	Hepatobiliary disorders	Primary	Primary	Not required	Primary	Primary; core endpoint
Hepatic injury	HEPATIC NECROSIS	Hepatobiliary disorders	Primary	Primary	Not required	Primary	Primary; core endpoint
Hepatic injury	HEPATITIS	Hepatobiliary disorders	Primary	Sensitivity	Primary	Sensitivity	Sensitivity; etiology ambiguous
Hepatic injury	HEPATITIS A	Infections and infestations	Excluded	Excluded	Not required	Excluded	Excluded; infectious mimic
Hepatic injury	HEPATITIS A ANTIBODY		Excluded	Excluded	Not required	Excluded	Excluded; lab only
Hepatic injury	HEPATITIS A ANTIBODY ABNORMAL		Excluded	Excluded	Not required	Excluded	Excluded; lab only
Hepatic injury	HEPATITIS A ANTIBODY NEGATIVE	not available in Canada extract	Excluded	Excluded	Not required	Excluded	Excluded; lab only
Hepatic injury	HEPATITIS A ANTIBODY POSITIVE		Excluded	Excluded	Not required	Excluded	Excluded; lab only

Endpoint	Candidate preferred term	MedDRA system organ class	Adjudicator 1	Adjudicator 2	Third adjudicator	Boundary-sensitivity role	Primary role and reason
Hepatic injury	HEPATITIS A ANTIGEN POSITIVE	not available in Canada extract	Excluded	Excluded	Not required	Excluded	Excluded; lab only
Hepatic injury	HEPATITIS A IMMUNISATION		Excluded	Excluded	Not required	Excluded	Excluded; indication or prophylaxis
Hepatic injury	HEPATITIS A POSITIVE	not available in Canada extract	Excluded	Excluded	Not required	Excluded	Excluded; lab only
Hepatic injury	HEPATITIS A VIRUS	not available in Canada extract	Excluded	Excluded	Not required	Excluded	Excluded; infectious mimic
Hepatic injury	HEPATITIS A VIRUS TEST	not available in Canada extract	Excluded	Excluded	Not required	Excluded	Excluded; lab only
Hepatic injury	HEPATITIS A VIRUS TEST POSITIVE		Excluded	Excluded	Not required	Excluded	Excluded; lab only
Hepatic injury	HEPATITIS ACUTE	Hepatobiliary disorders	Primary	Primary	Not required	Primary	Primary; core endpoint
Hepatic injury	HEPATITIS ALCOHOLIC		Excluded	Excluded	Not required	Excluded	Excluded; wrong endpoint or unrelated; metabolic or secondary cause
Hepatic injury	HEPATITIS B	Infections and infestations	Excluded	Excluded	Not required	Excluded	Excluded; infectious mimic
Hepatic injury	HEPATITIS B ANTIBODY	not available in Canada extract	Excluded	Excluded	Not required	Excluded	Excluded; lab only
Hepatic injury	HEPATITIS B ANTIBODY ABNORMAL	not available in Canada extract	Excluded	Excluded	Not required	Excluded	Excluded; lab only
Hepatic injury	HEPATITIS B ANTIBODY NEGATIVE		Excluded	Excluded	Not required	Excluded	Excluded; lab only
Hepatic injury	HEPATITIS B ANTIBODY POSITIVE	Investigations	Excluded	Excluded	Not required	Excluded	Excluded; lab only
Hepatic injury	HEPATITIS B ANTIGEN	not available in Canada extract	Excluded	Excluded	Not required	Excluded	Excluded; lab only
Hepatic injury	HEPATITIS B ANTIGEN POSITIVE		Excluded	Excluded	Not required	Excluded	Excluded; lab only
Hepatic injury	Hepatitis B core antibody	Investigations	Excluded	Excluded	Not required	Excluded	Excluded; lab only
Hepatic injury	Hepatitis B core antibody negative		Excluded	Excluded	Not required	Excluded	Excluded; lab only
Hepatic injury	HEPATITIS B CORE ANTIBODY POSITIVE	Investigations	Excluded	Excluded	Not required	Excluded	Excluded; lab only
Hepatic injury	HEPATITIS B CORE ANTIGEN	not available in Canada extract	Excluded	Excluded	Not required	Excluded	Excluded; lab only
Hepatic injury	HEPATITIS B CORE ANTIGEN POSITIVE		Excluded	Excluded	Not required	Excluded	Excluded; lab only
Hepatic injury	HEPATITIS B DNA ASSAY	not available in Canada extract	Excluded	Excluded	Not required	Excluded	Excluded; lab only
Hepatic injury	Hepatitis B DNA assay negative		Excluded	Excluded	Not required	Excluded	Excluded; lab only
Hepatic injury	HEPATITIS B DNA ASSAY POSITIVE		Excluded	Excluded	Not required	Excluded	Excluded; lab only
Hepatic injury	HEPATITIS B DNA DECREASED		Excluded	Excluded	Not required	Excluded	Excluded; lab only
Hepatic injury	HEPATITIS B DNA INCREASED		Excluded	Excluded	Not required	Excluded	Excluded; lab only
Hepatic injury	Hepatitis B e antibody negative		Excluded	Excluded	Not required	Excluded	Excluded; lab only
Hepatic injury	HEPATITIS B E ANTIBODY POSITIVE	not available in Canada extract	Excluded	Excluded	Not required	Excluded	Excluded; lab only
Hepatic injury	HEPATITIS B E ANTIGEN	not available in Canada extract	Excluded	Excluded	Not required	Excluded	Excluded; lab only
Hepatic injury	Hepatitis B e antigen negative		Excluded	Excluded	Not required	Excluded	Excluded; lab only
Hepatic injury	HEPATITIS B E ANTIGEN POSITIVE		Excluded	Excluded	Not required	Excluded	Excluded; lab only
Hepatic injury	Hepatitis B immunisation		Excluded	Excluded	Not required	Excluded	Excluded; indication or prophylaxis
Hepatic injury	HEPATITIS B POSITIVE	not available in Canada extract	Excluded	Excluded	Not required	Excluded	Excluded; lab only

Endpoint	Candidate preferred term	MedDRA system organ class	Adjudicator 1	Adjudicator 2	Third adjudicator	Boundary-sensitivity role	Primary role and reason
Hepatic injury	Hepatitis B reactivation		Excluded	Excluded	Not required	Excluded	Excluded; infectious mimic
Hepatic injury	Hepatitis B surface antibody	Investigations	Excluded	Excluded	Not required	Excluded	Excluded; lab only
Hepatic injury	Hepatitis B surface antibody negative		Excluded	Excluded	Not required	Excluded	Excluded; lab only
Hepatic injury	HEPATITIS B SURFACE ANTIBODY POSITIVE	Investigations	Excluded	Excluded	Not required	Excluded	Excluded; lab only
Hepatic injury	HEPATITIS B SURFACE ANTIGEN	not available in Canada extract	Excluded	Excluded	Not required	Excluded	Excluded; lab only
Hepatic injury	HEPATITIS B SURFACE ANTIGEN NEGATIVE		Excluded	Excluded	Not required	Excluded	Excluded; lab only
Hepatic injury	HEPATITIS B SURFACE ANTIGEN POSITIVE		Excluded	Excluded	Not required	Excluded	Excluded; lab only
Hepatic injury	HEPATITIS B TEST NEGATIVE	Investigations	Excluded	Excluded	Not required	Excluded	Excluded; lab only
Hepatic injury	HEPATITIS B VIRUS	not available in Canada extract	Excluded	Excluded	Not required	Excluded	Excluded; infectious mimic
Hepatic injury	HEPATITIS B VIRUS TEST	not available in Canada extract	Excluded	Excluded	Not required	Excluded	Excluded; lab only
Hepatic injury	HEPATITIS B VIRUS TEST POSITIVE	Investigations	Excluded	Excluded	Not required	Excluded	Excluded; lab only
Hepatic injury	HEPATITIS C	Infections and infestations	Excluded	Excluded	Not required	Excluded	Excluded; infectious mimic
Hepatic injury	HEPATITIS C ANTIBODY	not available in Canada extract	Excluded	Excluded	Not required	Excluded	Excluded; lab only
Hepatic injury	Hepatitis C antibody negative		Excluded	Excluded	Not required	Excluded	Excluded; lab only
Hepatic injury	HEPATITIS C ANTIBODY POSITIVE		Excluded	Excluded	Not required	Excluded	Excluded; lab only
Hepatic injury	Hepatitis C core antibody positive		Excluded	Excluded	Not required	Excluded	Excluded; lab only
Hepatic injury	HEPATITIS C POSITIVE	not available in Canada extract	Excluded	Excluded	Not required	Excluded	Excluded; lab only
Hepatic injury	HEPATITIS C RNA		Excluded	Excluded	Not required	Excluded	Excluded; lab only
Hepatic injury	Hepatitis C RNA decreased		Excluded	Excluded	Not required	Excluded	Excluded; lab only
Hepatic injury	HEPATITIS C RNA FLUCTUATION	not available in Canada extract	Excluded	Excluded	Not required	Excluded	Excluded; lab only
Hepatic injury	HEPATITIS C RNA INCREASED		Excluded	Excluded	Not required	Excluded	Excluded; lab only
Hepatic injury	HEPATITIS C RNA NEGATIVE		Excluded	Excluded	Not required	Excluded	Excluded; lab only
Hepatic injury	HEPATITIS C RNA POSITIVE		Excluded	Excluded	Not required	Excluded	Excluded; lab only
Hepatic injury	HEPATITIS C TEST NEGATIVE	not available in Canada extract	Excluded	Excluded	Not required	Excluded	Excluded; lab only
Hepatic injury	HEPATITIS C VIRUS	not available in Canada extract	Excluded	Excluded	Not required	Excluded	Excluded; infectious mimic
Hepatic injury	Hepatitis C virus core antigen	not available in Canada extract	Excluded	Excluded	Not required	Excluded	Excluded; lab only
Hepatic injury	HEPATITIS C VIRUS TEST	not available in Canada extract	Excluded	Excluded	Not required	Excluded	Excluded; lab only
Hepatic injury	HEPATITIS C VIRUS TEST POSITIVE	Investigations	Excluded	Excluded	Not required	Excluded	Excluded; lab only
Hepatic injury	HEPATITIS CHOLESTATIC	Hepatobiliary disorders	Primary	Primary	Not required	Primary	Primary; core endpoint
Hepatic injury	HEPATITIS CHRONIC ACTIVE	Hepatobiliary disorders	Primary	Excluded	Sensitivity	Sensitivity	Sensitivity; chronic or underlying disease
Hepatic injury	HEPATITIS CHRONIC PERSISTENT		Primary	Excluded	Sensitivity	Sensitivity	Sensitivity; chronic or underlying disease
Hepatic injury	HEPATITIS D		Excluded	Excluded	Not required	Excluded	Excluded; infectious mimic
Hepatic injury	Hepatitis D antibody positive	not available in Canada extract	Excluded	Excluded	Not required	Excluded	Excluded; lab only
Hepatic injury	Hepatitis D RNA positive	not available in Canada extract	Excluded	Excluded	Not required	Excluded	Excluded; lab only

Endpoint	Candidate preferred term	MedDRA system organ class	Adjudicator 1	Adjudicator 2	Third adjudicator	Boundary-sensitivity role	Primary role and reason
Hepatic injury	HEPATITIS D VIRUS TEST POSITIVE	not available in Canada extract	Excluded	Excluded	Not required	Excluded	Excluded; lab only
Hepatic injury	HEPATITIS E		Excluded	Excluded	Not required	Excluded	Excluded; infectious mimic
Hepatic injury	HEPATITIS E ANTIBODY	not available in Canada extract	Excluded	Excluded	Not required	Excluded	Excluded; lab only
Hepatic injury	HEPATITIS E ANTIBODY POSITIVE	not available in Canada extract	Excluded	Excluded	Not required	Excluded	Excluded; lab only
Hepatic injury	HEPATITIS E ANTIGEN	not available in Canada extract	Excluded	Excluded	Not required	Excluded	Excluded; lab only
Hepatic injury	Hepatitis E antigen negative	not available in Canada extract	Excluded	Excluded	Not required	Excluded	Excluded; lab only
Hepatic injury	HEPATITIS E ANTIGEN POSITIVE	not available in Canada extract	Excluded	Excluded	Not required	Excluded	Excluded; lab only
Hepatic injury	Hepatitis E virus test positive		Excluded	Excluded	Not required	Excluded	Excluded; lab only
Hepatic injury	HEPATITIS FULMINANT	Hepatobiliary disorders	Primary	Primary	Not required	Primary	Primary; core endpoint
Hepatic injury	HEPATITIS G	not available in Canada extract	Excluded	Excluded	Not required	Excluded	Excluded; infectious mimic
Hepatic injury	HEPATITIS GRANULOMATOUS	not available in Canada extract	Primary	Sensitivity	Primary	Primary	Sensitivity; etiology ambiguous
Hepatic injury	Hepatitis immunisation	not available in Canada extract	Excluded	Excluded	Not required	Excluded	Excluded; indication or prophylaxis
Hepatic injury	HEPATITIS INFECTIOUS	not available in Canada extract	Excluded	Excluded	Not required	Excluded	Excluded; infectious mimic
Hepatic injury	HEPATITIS INFECTIOUS MONONUCLEOSIS		Excluded	Excluded	Not required	Excluded	Excluded; infectious mimic
Hepatic injury	Hepatitis mumps	not available in Canada extract	Excluded	Excluded	Not required	Excluded	Excluded; infectious mimic
Hepatic injury	HEPATITIS NEONATAL	not available in Canada extract	Excluded	Excluded	Not required	Sensitivity	Excluded; pregnancy fetal perinatal
Hepatic injury	HEPATITIS NON-A NON-B		Excluded	Excluded	Not required	Excluded	Excluded; infectious mimic
Hepatic injury	HEPATITIS NON-A NON-B NON-C	not available in Canada extract	Excluded	Excluded	Not required	Excluded	Excluded; infectious mimic
Hepatic injury	HEPATITIS POST TRANSFUSION		Excluded	Excluded	Not required	Excluded	Excluded; infectious mimic
Hepatic injury	HEPATITIS SYPHILITIC	not available in Canada extract	Excluded	Excluded	Not required	Excluded	Excluded; infectious mimic
Hepatic injury	HEPATITIS TOXIC	Hepatobiliary disorders	Primary	Primary	Not required	Primary	Primary; core endpoint
Hepatic injury	HEPATITIS VIRAL	Infections and infestations	Excluded	Excluded	Not required	Excluded	Excluded; infectious mimic
Hepatic injury	Hepatitis viral test	not available in Canada extract	Excluded	Excluded	Not required	Excluded	Excluded; lab only
Hepatic injury	Hepatitis viral test positive	not available in Canada extract	Excluded	Excluded	Not required	Excluded	Excluded; lab only
Hepatic injury	HEPATITIS VIRUS-ASSOCIATED NEPHROPATHY	not available in Canada extract	Excluded	Excluded	Not required	Excluded	Excluded; wrong endpoint or unrelated
Hepatic injury	HEPATOTOXICITY	Hepatobiliary disorders	Primary	Primary	Not required	Primary	Primary; core endpoint
Hepatic injury	HERPES SIMPLEX HEPATITIS		Excluded	Excluded	Not required	Excluded	Excluded; infectious mimic
Hepatic injury	HYPERBILIRUBINAEMIA	Hepatobiliary disorders	Sensitivity	Sensitivity	Not required	Sensitivity	Sensitivity; lab only; nonspecific broad
Hepatic injury	HYPERBILIRUBINAEMIA NEONATAL	Hepatobiliary disorders	Excluded	Excluded	Not required	Sensitivity	Excluded; pregnancy fetal perinatal
Hepatic injury	Immune-mediated cholestasis		Primary	Primary	Not required	Primary	Primary; core endpoint
Hepatic injury	Immune-mediated hepatitis	Hepatobiliary disorders	Primary	Primary	Not required	Primary	Primary; core endpoint
Hepatic injury	ISCHAEMIC HEPATITIS	Hepatobiliary disorders	Sensitivity	Excluded	Sensitivity	Sensitivity	Sensitivity; secondary hemodynamic context
Hepatic injury	JAUNDICE	Hepatobiliary disorders	Primary	Primary	Not required	Primary	Primary; core endpoint

Endpoint	Candidate preferred term	MedDRA system organ class	Adjudicator 1	Adjudicator 2	Third adjudicator	Boundary-sensitivity role	Primary role and reason
Hepatic injury	JAUNDICE ACHOLURIC	not available in Canada extract	Excluded	Excluded	Not required	Sensitivity	Excluded; wrong endpoint or unrelated
Hepatic injury	JAUNDICE CHOLESTATIC	Hepatobiliary disorders	Primary	Primary	Not required	Primary	Primary; core endpoint
Hepatic injury	JAUNDICE EXTRAHEPATIC OBSTRUCTIVE	not available in Canada extract	Sensitivity	Excluded	Excluded	Sensitivity	Excluded; wrong endpoint or obstructive context
Hepatic injury	JAUNDICE HEPATOCELLULAR	Hepatobiliary disorders	Primary	Primary	Not required	Primary	Primary; core endpoint
Hepatic injury	JAUNDICE NEONATAL	Pregnancy, puerperium and perinatal conditions	Excluded	Excluded	Not required	Sensitivity	Excluded; pregnancy fetal perinatal
Hepatic injury	LIVER FUNCTION TEST ABNORMAL	Investigations	Sensitivity	Sensitivity	Not required	Sensitivity	Sensitivity; lab only
Hepatic injury	LIVER INJURY	Hepatobiliary disorders	Primary	Primary	Not required	Primary	Primary; core endpoint
Hepatic injury	LUPUS HEPATITIS	not available in Canada extract	Sensitivity	Excluded	Sensitivity	Excluded	Sensitivity; disease or immune mimic
Hepatic injury	Mitochondrial aspartate aminotransferase increased	not available in Canada extract	Sensitivity	Sensitivity	Not required	Sensitivity	Sensitivity; lab only
Hepatic injury	MIXED HEPATOCELLULAR-CHOLESTATIC INJURY	not available in Canada extract	Primary	Primary	Not required	Primary	Primary; core endpoint
Hepatic injury	MIXED LIVER INJURY	Hepatobiliary disorders	Primary	Primary	Not required	Primary	Primary; core endpoint
Hepatic injury	NEONATAL CHOLESTASIS	Hepatobiliary disorders	Excluded	Excluded	Not required	Sensitivity	Excluded; pregnancy fetal perinatal
Hepatic injury	Progressive familial intrahepatic cholestasis	not available in Canada extract	Excluded	Excluded	Not required	Excluded	Excluded; congenital or developmental
Hepatic injury	RADIATION HEPATITIS	not available in Canada extract	Excluded	Excluded	Not required	Excluded	Excluded; wrong endpoint or unrelated
Hepatic injury	SUBACUTE HEPATIC FAILURE		Primary	Primary	Not required	Primary	Primary; core endpoint
Hepatic injury	Suspected drug-induced liver injury		Primary	Primary	Not required	Primary	Primary; core endpoint
Hepatic injury	TRANSAMINASES INCREASED	Investigations	Sensitivity	Sensitivity	Not required	Sensitivity	Sensitivity; lab only
Hepatic injury	TRAUMATIC LIVER INJURY	Injury, poisoning and procedural complications	Excluded	Excluded	Not required	Excluded	Excluded; wrong endpoint or unrelated
Hepatic injury	URINE BILIRUBIN INCREASED		Sensitivity	Sensitivity	Not required	Sensitivity	Sensitivity; lab only
Hepatic injury	VIRAL HEPATITIS CARRIER		Excluded	Excluded	Not required	Excluded	Excluded; infectious mimic
Hepatic injury	Withdrawal hepatitis	not available in Canada extract	Primary	Sensitivity	Primary	Sensitivity	Sensitivity; withdrawal context
QT / proarrhythmia	CARDIAC ARREST	Cardiac disorders	Sensitivity	Sensitivity	Not required	Sensitivity	Sensitivity; nonspecific broad
QT / proarrhythmia	CARDIAC ARREST NEONATAL	Cardiac disorders	Excluded	Excluded	Not required	Sensitivity	Excluded; pregnancy fetal perinatal
QT / proarrhythmia	ELECTROCARDIOGRAM QT CORRECTED INTERVAL PROLONGED	not available in Canada extract	Primary	Primary	Not required	Primary	Primary; core endpoint
QT / proarrhythmia	ELECTROCARDIOGRAM QT PROLONGED	Investigations	Primary	Primary	Not required	Primary	Primary; core endpoint
QT / proarrhythmia	Foetal cardiac arrest		Excluded	Excluded	Not required	Excluded	Excluded; pregnancy fetal perinatal
QT / proarrhythmia	LONG QT SYNDROME	Cardiac disorders	Sensitivity	Sensitivity	Not required	Primary	Sensitivity; nonspecific broad
QT / proarrhythmia	LONG QT SYNDROME CONGENITAL	not available in Canada extract	Excluded	Excluded	Not required	Excluded	Excluded; congenital or developmental
QT / proarrhythmia	Post cardiac arrest syndrome		Excluded	Excluded	Not required	Sensitivity	Excluded; wrong endpoint or unrelated
QT / proarrhythmia	SUDDEN CARDIAC DEATH	General disorders and administration site conditions	Sensitivity	Sensitivity	Not required	Sensitivity	Sensitivity; nonspecific broad

Endpoint	Candidate preferred term	MedDRA system organ class	Adjudicator 1	Adjudicator 2	Third adjudicator	Boundary-sensitivity role	Primary role and reason
QT / proarrhythmia	TORSADE DE POINTES	Cardiac disorders	Primary	Primary	Not required	Primary	Primary; core endpoint
QT / proarrhythmia	VENTRICULAR ARRHYTHMIA	Cardiac disorders	Primary	Primary	Not required	Primary	Primary; core endpoint
QT / proarrhythmia	VENTRICULAR FIBRILLATION	Cardiac disorders	Primary	Primary	Not required	Primary	Primary; core endpoint
QT / proarrhythmia	VENTRICULAR TACHYCARDIA	Cardiac disorders	Primary	Primary	Not required	Primary	Primary; core endpoint
Seizure	Acute encephalitis with refractory, repetitive partial seizures	not available in Canada extract	Sensitivity	Excluded	Sensitivity	Excluded	Sensitivity; infectious or syndromic context
Seizure	ALCOHOLIC SEIZURE		Sensitivity	Excluded	Sensitivity	Excluded	Sensitivity; substance or secondary context
Seizure	Atypical absence seizure	not available in Canada extract	Primary	Primary	Not required	Primary	Primary; core endpoint
Seizure	AUTONOMIC SEIZURE		Primary	Primary	Not required	Primary	Primary; core endpoint
Seizure	Change in seizure presentation	not available in Canada extract	Sensitivity	Sensitivity	Not required	Sensitivity	Sensitivity; nonspecific broad
Seizure	CLONIC CONVULSION		Primary	Primary	Not required	Primary	Primary; core endpoint
Seizure	COMPLEX PARTIAL SEIZURES	not available in Canada extract	Primary	Primary	Not required	Primary	Primary; core endpoint
Seizure	CONVULSION	not available in Canada extract	Primary	Primary	Not required	Primary	Primary; core endpoint
Seizure	CONVULSION IN CHILDHOOD	not available in Canada extract	Primary	Primary	Not required	Primary	Primary; core endpoint
Seizure	CONVULSION NEONATAL	not available in Canada extract	Primary	Primary	Not required	Primary	Primary; core endpoint
Seizure	CONVULSION PROPHYLAXIS	not available in Canada extract	Excluded	Excluded	Not required	Excluded	Excluded; indication or prophylaxis
Seizure	Faciobrachial dystonic seizure		Primary	Primary	Not required	Sensitivity	Primary; core endpoint
Seizure	FEBRILE CONVULSION	Nervous system disorders	Sensitivity	Sensitivity	Not required	Sensitivity	Sensitivity; metabolic or secondary cause
Seizure	Febrile status epilepticus	not available in Canada extract	Sensitivity	Sensitivity	Not required	Sensitivity	Sensitivity; metabolic or secondary cause
Seizure	Gelastic seizure		Primary	Primary	Not required	Primary	Primary; core endpoint
Seizure	Generalised onset non-motor seizure	not available in Canada extract	Primary	Primary	Not required	Primary	Primary; core endpoint
Seizure	Generalised tonic-clonic seizure	Nervous system disorders	Primary	Primary	Not required	Primary	Primary; core endpoint
Seizure	GRAND MAL CONVULSION	not available in Canada extract	Primary	Primary	Not required	Primary	Primary; core endpoint
Seizure	Hemiclonic seizure	not available in Canada extract	Primary	Primary	Not required	Primary	Primary; core endpoint
Seizure	HYPERGLYCAEMIC SEIZURE	not available in Canada extract	Sensitivity	Sensitivity	Not required	Sensitivity	Sensitivity; metabolic or secondary cause
Seizure	Hypocalcaemic seizure	not available in Canada extract	Sensitivity	Sensitivity	Not required	Sensitivity	Sensitivity; metabolic or secondary cause
Seizure	HYPOGLYCAEMIC SEIZURE	Nervous system disorders	Sensitivity	Sensitivity	Not required	Sensitivity	Sensitivity; metabolic or secondary cause
Seizure	Hyponatraemic seizure	Nervous system disorders	Sensitivity	Sensitivity	Not required	Sensitivity	Sensitivity; metabolic or secondary cause
Seizure	Migraine-triggered seizure		Sensitivity	Sensitivity	Not required	Sensitivity	Sensitivity; metabolic or secondary cause
Seizure	Neonatal epileptic seizure	not available in Canada extract	Primary	Primary	Not required	Primary	Primary; core endpoint
Seizure	Neonatal seizure	Nervous system disorders	Primary	Primary	Not required	Primary	Primary; core endpoint
Seizure	New onset refractory status epilepticus		Primary	Primary	Not required	Primary	Primary; core endpoint
Seizure	PARTIAL SEIZURES	Nervous system disorders	Primary	Primary	Not required	Primary	Primary; core endpoint
Seizure	PARTIAL SEIZURES WITH SECONDARY GENERALISATION	Nervous system disorders	Primary	Primary	Not required	Primary	Primary; core endpoint

Endpoint	Candidate preferred term	MedDRA system organ class	Adjudicator 1	Adjudicator 2	Third adjudicator	Boundary-sensitivity role	Primary role and reason
Seizure	Photosensitive seizure	not available in Canada extract	Primary	Primary	Not required	Primary	Primary; core endpoint
Seizure	Post stroke seizure		Sensitivity	Sensitivity	Not required	Sensitivity	Sensitivity; metabolic or secondary cause
Seizure	Psychogenic seizure	Nervous system disorders	Excluded	Excluded	Not required	Excluded	Excluded; wrong endpoint or unrelated
Seizure	Seizure	Nervous system disorders	Primary	Primary	Not required	Primary	Primary; core endpoint
Seizure	SEIZURE ANOXIC	Nervous system disorders	Sensitivity	Sensitivity	Not required	Sensitivity	Sensitivity; metabolic or secondary cause
Seizure	SEIZURE CLUSTER	Nervous system disorders	Primary	Primary	Not required	Primary	Primary; core endpoint
Seizure	SEIZURE LIKE PHENOMENA	Nervous system disorders	Sensitivity	Sensitivity	Not required	Sensitivity	Sensitivity; nonspecific broad
Seizure	Seizure prophylaxis	not available in Canada extract	Excluded	Excluded	Not required	Excluded	Excluded; indication or prophylaxis
Seizure	SIMPLE PARTIAL SEIZURES		Primary	Primary	Not required	Primary	Primary; core endpoint
Seizure	STATUS EPILEPTICUS	Nervous system disorders	Primary	Primary	Not required	Primary	Primary; core endpoint
Seizure	Tardive seizure	not available in Canada extract	Primary	Primary	Not required	Primary	Primary; core endpoint
Seizure	TONIC CONVULSION	Nervous system disorders	Primary	Primary	Not required	Primary	Primary; core endpoint

Supplementary Table S15. Endpoint-definition coverage sensitivity. Primary and sensitivity-only preferred terms are counted separately. Reaction-row counts quantify the additional coded-event volume available under the broader sensitivity role. Canada report counts are not additive because one report can contain preferred terms from both roles.

Endpoint	Source	Primary PTs	Sensitivity PTs	Primary reaction rows	Sensitivity reaction rows	Canada reports with primary PT	Canada reports with sensitivity PT
Acute kidney injury	FAERS/AERS	3	9	265,364	443,119	–	–
Acute kidney injury	Canada Vigilance	3	9	6,326	17,008	276	398
Anaphylaxis	FAERS/AERS	4	1	101,309	3,666	–	–
Anaphylaxis	Canada Vigilance	4	1	10,508	236	911	121
Hepatic injury	FAERS/AERS	25	22	267,950	333,751	–	–
Hepatic injury	Canada Vigilance	25	22	9,663	16,078	416	668
QT/proarrhythmia	FAERS/AERS	6	3	95,341	107,163	–	–
QT/proarrhythmia	Canada Vigilance	6	3	3,547	3,233	197	180
Seizure	FAERS/AERS	25	13	280,474	6,199	–	–
Seizure	Canada Vigilance	25	13	13,364	417	1,597	135

Supplementary Table S16. Pair-label sensitivity to endpoint-definition choice. The same FAERS source-signal rule, Canada manufacturer-screened target rule, medicinal-product eligibility rule, and 40-pair anaphylaxis reservation list were applied to the primary and boundary-sensitivity definitions. The boundary-sensitivity and primary PT counts were 6/3 for acute kidney injury, 4/4 for anaphylaxis, 29/25 for hepatic injury, 7/6 for QT/proarrhythmia, and 24/25 for seizure, respectively. Candidate-level role changes occurred in all five endpoint families and are reported in Supplementary Table S14.

Definition	Positive	Negative	Borderline	Ambiguous	Other observable	Reserved
Primary five-endpoint definition	147	481	155	20	0	18
Boundary-sensitivity definition	150	481	157	20	0	18

Supplementary Table S16 (continued). Endpoint- and period-specific label counts under the two endpoint-definition sets.

Definition	Period	Endpoint	Positive	Negative	Borderline
Primary five-endpoint definition	Future	Acute kidney injury	7	60	16
Primary five-endpoint definition	Future	Anaphylaxis	19	13	6
Primary five-endpoint definition	Future	Hepatic injury	5	92	34
Primary five-endpoint definition	Future	QT/proarrhythmia	13	68	12
Primary five-endpoint definition	Future	Seizure	18	17	15
Primary five-endpoint definition	Historical	Acute kidney injury	25	62	21
Primary five-endpoint definition	Historical	Anaphylaxis	15	11	16
Primary five-endpoint definition	Historical	Hepatic injury	12	64	10
Primary five-endpoint definition	Historical	QT/proarrhythmia	9	56	13
Primary five-endpoint definition	Historical	Seizure	24	38	12
Boundary-sensitivity definition	Future	Acute kidney injury	7	57	15
Boundary-sensitivity definition	Future	Anaphylaxis	19	13	6
Boundary-sensitivity definition	Future	Hepatic injury	5	93	35
Boundary-sensitivity definition	Future	QT/proarrhythmia	13	67	12
Boundary-sensitivity definition	Future	Seizure	18	17	15
Boundary-sensitivity definition	Historical	Acute kidney injury	28	62	22
Boundary-sensitivity definition	Historical	Anaphylaxis	15	11	16
Boundary-sensitivity definition	Historical	Hepatic injury	12	64	11
Boundary-sensitivity definition	Historical	QT/proarrhythmia	9	59	13
Boundary-sensitivity definition	Historical	Seizure	24	38	12

Supplementary Table S17. Public age-field availability and pediatric-eligibility handling. Exact numeric-age completeness is reported for FAERS/AERS and Canada Vigilance. Decade-coded FAERS ages were classified as non-exact in this completeness summary but were converted to years when they unambiguously satisfied the pediatric eligibility rule. Missing age was not imputed for pediatric cohort membership. JADER records categorical age bands rather than comparable numeric age, so exact numeric-age completeness was not calculated.

Source	Public age field	Report rows	Numerically usable age	Numeric age missing	Missing (%)	Use in pediatric eligibility
Canada Vigilance	Exact numeric age and age unit	1,240,378	955,454	284,924	23.0	Reports without an age convertible under the benchmark rule were not assigned pediatric status.
FAERS/AERS	Exact numeric age and age unit	24,389,966	14,134,920	10,255,046	42.0	Reports without an age convertible under the benchmark rule were not assigned pediatric status.
JADER	Public categorical age band	1,032,027	–	–	–	Not applicable to the numeric pediatric label definition.

Supplementary Table S18. Evaluation-regime reconciliation. (A) Primary cross-validation. Primary cross-validation and the secondary feature-family evaluations answer different questions and should not be compared as estimates from the same split. Absolute AUPRC is reported for each listed model or feature set.

Evaluation regime	Comparator or feature set	Mean AUPRC	Difference from full FAERS	Uncertainty or variability	Interpretation
Primary five-fold endpoint/period/label-stratified cross-validation	EBM	0.751	–	–	Primary within-observed-product benchmark evaluation; not a powered temporal-generalization test.
Primary five-fold endpoint/period/label-stratified cross-validation	Elastic-net logistic regression	0.767	–	–	Primary within-observed-product benchmark evaluation; not a powered temporal-generalization test.
Primary five-fold endpoint/period/label-stratified cross-validation	Endpoint-conditioned co-report	0.753	–	–	Primary within-observed-product benchmark evaluation; not a powered temporal-generalization test.
Primary five-fold endpoint/period/label-stratified cross-validation	LightGBM	0.774	–	–	Primary within-observed-product benchmark evaluation; not a powered temporal-generalization test.
Primary five-fold endpoint/period/label-stratified cross-validation	LightGBM + chemical structure	0.795	–	+0.042 vs endpoint-conditioned co-report	Full-label estimate from the label-efficiency analysis under the primary cross-validation design.

Supplementary Table S18 (continued). (B) Repeated drug-held-out partitions. No medicinal-product identity was shared between training and test partitions. Variability is summarized by the standard deviation across 30 partitions.

Evaluation regime	Comparator or feature set	Mean AUPRC	Difference from full FAERS	Uncertainty or variability	Interpretation
Thirty repeated drug-held-out partitions	Full FAERS source-signal baseline	0.764	+0.000	Partition SD 0.014	Secondary feature-family robustness analysis with no drug identity shared between training and test partitions.
Thirty repeated drug-held-out partitions	Full FAERS + drug class	0.787	+0.023	Partition SD 0.016	Secondary feature-family robustness analysis with no drug identity shared between training and test partitions.
Thirty repeated drug-held-out partitions	Full FAERS + chemical structure	0.796	+0.032	Partition SD 0.019	Secondary feature-family robustness analysis with no drug identity shared between training and test partitions.
Thirty repeated drug-held-out partitions	Full FAERS + structure + drug class	0.800	+0.036	Partition SD 0.018	Secondary feature-family robustness analysis with no drug identity shared between training and test partitions.
Thirty repeated drug-held-out partitions	Full FAERS + structure + drug class + target/pathway	0.801	+0.037	Partition SD 0.020	Secondary feature-family robustness analysis with no drug identity shared between training and test partitions.

Supplementary Table S18 (continued). (C) Unseen-scaffold partitions and fixed replication analyses. The unseen-scaffold regime was restricted to 275 medicinal-product identities with valid Bemis–Murcko scaffolds, so its absolute AUPRC is not directly comparable with the 319-identity drug-held-out regimes. Repeated-partition rows show between-partition standard deviations; the fixed replications show clustered paired-difference intervals.

Evaluation regime	Comparator or feature set	Mean AUPRC	Difference from full FAERS	Uncertainty or variability	Interpretation
Thirty unseen-scaffold partitions	Full FAERS source-signal baseline	0.702	+0.000	Partition SD 0.019	Restricted analysis with no medicinal-product identity or Bemis–Murcko scaffold shared between training and test partitions.
Thirty unseen-scaffold partitions	Full FAERS + chemical structure	0.699	-0.003	Partition SD 0.024	Chemical structure did not improve performance when test scaffolds were absent from training.
Thirty unseen-scaffold partitions	Full FAERS + structure + drug class	0.698	-0.005	Partition SD 0.023	Drug class did not restore the structure increment under unseen-scaffold evaluation.
Feature-family standard-cross-validation replication	Full FAERS source-signal baseline (LightGBM)	0.778	+0.000	–	Fixed standard-cross-validation replication paired with the drug-held-out feature-family check; not the primary benchmark estimate.
Feature-family standard-cross-validation replication	Full FAERS + structure + drug class (LightGBM)	0.797	+0.018	Cluster-bootstrap 95% CI -0.020 to +0.058	The interval included zero; this replication is reported alongside the separate drug-held-out check.
Single drug-held-out replication	Full FAERS source-signal baseline	0.770	+0.000	–	One drug-held-out partition used for the clustered paired-difference replication.
Single drug-held-out replication	Full FAERS + structure + drug class	0.797	+0.027	Cluster-bootstrap 95% CI -0.002 to +0.058	The interval included zero; this supports treating the feature-family finding as secondary.

Supplementary Table S19. Signal and performance metric definitions and formulas. For the drug–event table, a is the number of reports containing both product and endpoint, b product reports without the endpoint, c endpoint reports without the product, d reports with neither, $n = a+b+c+d$, and $x' = x + 0.5$ denotes the continuity-corrected cell count. For performance metrics, $y_i \in \{0, 1\}$ and p_i are the observed label and predicted probability for row i.

Metric	Operational formula and interpretation
Reporting odds ratio lower 95% confidence bound	$\mathrm{ROR} = a'd'/(b'c')$; $\mathrm{SE}\{\log(\mathrm{ROR})\} = \sqrt{1/a' + 1/b' + 1/c' + 1/d'}$; $\mathrm{ROR}_{L95} = \exp[\log(\mathrm{ROR}) - 1.96\,\mathrm{SE}]$. Higher values indicate stronger disproportionate co-reporting.
Proportional reporting ratio	$\mathrm{PRR} = [a'/(a' + b')]/[c'/(c' + d')]$. Values above one indicate that the endpoint occupies a larger fraction of reports for the product than for other products.
Pearson chi-square statistic	$\chi^2 = n(ad - bc)^2/[(a + b)(c + d)(a + c)(b + d)]$; set to zero when the denominator is zero. Higher values indicate stronger departure from independence.
Information-component-like score	With $E = (a + b)(a + c)/n$, $\mathrm{IC}_{\mathrm{like}} = \log_2[(a + 0.5)/(E + 0.5)]$. Positive values indicate observed co-reporting above the independence expectation.
Area under the precision-recall curve (AUPRC)	Operationally computed as average precision, $\mathrm{AP} = \sum_k (R_k - R_{k-1})P_k$, where P_k and R_k are precision and recall at successive score thresholds. Higher values indicate better ranking of positive rows and depend on outcome prevalence.
Area under the receiver-operating characteristic curve (AUROC)	$\Pr(s^+ > s^-) + 0.5 \Pr(s^+ = s^-)$, where s^+ and s^- are scores for randomly selected positive and negative rows. Higher values indicate better discrimination.
Brier score	$\mathrm{Brier} = N^{-1} \sum_{i=1}^{N} (p_i - y_i)^2$. Lower values indicate more accurate probabilistic predictions.
Calibration intercept and slope	Estimated from $\mathrm{logit}\{\Pr(Y_i = 1)\} = \alpha + \beta\,\mathrm{logit}(p_i)$. Ideal calibration has intercept $\alpha = 0$ and slope $\beta = 1$.
Expected calibration error (10 bins)	$\mathrm{ECE} = \sum_{m=1}^{10} (\lvert B_m\rvert/N)\,\lvert\bar{y}_m - \bar{p}_m\rvert$, using ten equal-width probability bins B_m. Lower values indicate closer agreement between predicted and observed event fractions.
Precision at a fixed triage budget	$\mathrm{Precision@}K = K^{-1} \sum_{i \in \mathrm{Top}\ K} y_i$. This is the positive fraction among the K highest-ranked rows.
Top-decile enrichment	$\mathrm{Enrichment}_{10\%} = \mathrm{Precision@}\lceil 0.1N \rceil/\bar{y}$. Values above one indicate enrichment over the overall positive fraction $\bar{y}$.

Supplementary Table S20. Temporal-window selection and medicinal-product mapping coverage. The historical start was chosen before label construction using an 80% quarterly identity-mapping coverage floor. The future boundary was aligned with the pre-2021 cutoff for dated pharmacological feature sources.

Window	Analysis role	Weighted coverage (%)	Minimum quarter (%)	Maximum quarter (%)	Rationale
2012Q3-2014Q2 transition segment	Sensitivity/descriptive only	–	65.1	73.5	Several quarters remained below the prespecified 80% medicinal-product identity-mapping coverage floor.
2014Q3-2020Q4 historical period	Primary historical period	88.4	83.2	90.8	Every quarter met the 80% coverage floor; the period ended before the prespecified 2021 future boundary.
2021Q1 onward	Future-period stratum	89.9	87.4	91.7	Aligned the future-period boundary with the pre-2021 knowledge cutoff used for dated target and pathway features.

Supplementary Table S21. Characteristics of reports with and without exact numerically usable age information. Selected categories, counts, and column percentages are shown for unfiltered FAERS/AERS and Canada Vigilance reports; the complete category-level table is provided as a source-data file. Standardized differences summarize between-group imbalance in these complete public extracts.

Source	Characteristic	Category	Age available, n (%)	Age missing, n (%)	Std. difference
Canada Vigilance	Manufacturer authorization-holder number present	Yes	682,913 (71.5)	237,987 (83.5)	-0.292
	Report type	Spontaneous	529,831 (55.5)	150,279 (52.7)	+0.054
		Study	387,635 (40.6)	109,216 (38.3)	+0.046
		Published	26,472 (2.8)	14,896 (5.2)	-0.126
		Unknown	550 (0.1)	2,367 (0.8)	-0.116
	Reporter source	MAH	606,579 (63.5)	226,000 (79.3)	-0.356
		Community	120,664 (12.6)	16,226 (5.7)	+0.242
		Hospital	113,772 (11.9)	8,161 (2.9)	+0.351
		Clinical Study	90,330 (9.5)	21,762 (7.6)	+0.065
		Missing	12,320 (1.3)	4,886 (1.7)	-0.035
	Reporting year	Mean (SD)	2013.6 (12.9)	2014.5 (11.8)	-0.072
	Serious report	Yes	662,738 (69.4)	185,774 (65.2)	+0.089
	Sex	Female	575,348 (60.2)	141,391 (49.6)	+0.214
		Male	367,770 (38.5)	92,085 (32.3)	+0.129
		Missing or unknown	12,336 (1.3)	51,448 (18.1)	-0.591
FAERS/ AERS	Manufacturer report number present	Yes	13,304,766 (94.1)	10,011,760 (97.6)	-0.177
	Reporter country	United States	8,541,495 (60.4)	7,476,022 (72.9)	-0.267
		Missing	333,955 (2.4)	151,198 (1.5)	+0.065
	Reporter occupation	Consumer (CN)	5,293,021 (37.4)	5,327,948 (52.0)	-0.295
		Physician (MD)	3,742,929 (26.5)	1,863,930 (18.2)	+0.200
		Other health professional (HP)	1,418,750 (10.0)	805,676 (7.9)	+0.076
		Pharmacist (PH)	934,130 (6.6)	491,800 (4.8)	+0.078
		Missing	850,010 (6.0)	498,041 (4.9)	+0.051
	Reporting year	Mean (SD)	2017.6 (5.5)	2018.0 (5.2)	-0.072
	Serious report	Yes	9,429,599 (66.7)	5,324,455 (51.9)	+0.305
	Sex	Female	8,273,911 (58.5)	4,581,925 (44.7)	+0.280

Source	Characteristic	Category	Age available, n (%)	Age missing, n (%)	Std. difference
		Male	5,557,030 (39.3)	2,896,779 (28.2)	+0.236
		Missing or unknown	294,760 (2.1)	2,675,819 (26.1)	-0.735
		Other or unspecified	9,219 (0.1)	100,523 (1.0)	-0.122

Top-20 report-level term overlap

Canada Vigilance, Reaction PT: 17/20 shared; Jaccard=0.739

Canada Vigilance, Suspect ingredient: 13/20 shared; Jaccard=0.481

FAERS/AERS, Reaction PT: 16/20 shared; Jaccard=0.667

FAERS/AERS, Suspect ingredient: 8/20 shared; Jaccard=0.250

Supplementary Table S22. Source processing and benchmark-row accounting. Panel A summarizes source-specific processing stages. Stages with different time windows or units are not interpreted as a single sequential attrition series. Panel B accounts for all 18,635 candidate drug–endpoint–period rows before construction of the supervised and anaphylaxis analysis sets.

Panel A. Report-level source processing

Source	Processing stage	Count	Unit	Scope	Interpretation
FAERS/AERS	Parsed public reports	24,389,966	reports	2004Q1-2026Q1 source extraction	Quarterly DEMO tables parsed by explicit headers.
FAERS/AERS	After public deletion lists	24,388,883	reports	2004Q1-2026Q1 source extraction	1,083 report rows listed for deletion were removed.
FAERS/AERS	Exact numeric age available	14,134,920	reports	2004Q1-2026Q1 source extraction	Age availability is not the same as pediatric eligibility.
FAERS/AERS	Pediatric serious reports	548,101	report-quarter contexts	all extracted quarters	Age younger than 18 years plus a serious outcome code.
FAERS/AERS	Resolved suspect active ingredient	359,301	reports	2014Q3-2024Q4 benchmark periods	Mapped report universe used to construct source reporting statistics; scope changes at this stage.
Canada Vigilance	Parsed public reports	1,240,378	reports	public extract through 2026-01-31	Full reports table.
Canada Vigilance	Exact numeric age available	955,454	reports	public extract through 2026-01-31	Age availability is not the same as pediatric eligibility.
Canada Vigilance	Pediatric reports	55,339	reports	public extract through 2026-01-31	Reported age convertible to younger than 18 years.
Canada Vigilance	Pediatric serious reports	36,213	reports	public extract through 2026-01-31	Pediatric reports carrying the public serious-report indicator.
Canada Vigilance	Resolved suspect active ingredient before manufacturer screening	20,906	reports	2014Q3-2024Q4 benchmark periods	Mapped report universe before detectable manufacturer cross-filing removal; scope changes at this stage.
Canada Vigilance	After detectable manufacturer cross-filing screening	19,302	reports	2014Q3-2024Q4 benchmark periods	Only cross-filings detectable from public manufacturer identifiers were removed.
JADER	Parsed public reports	1,032,027	reports	2004-2025 descriptive extract	Third-country descriptive branch only.
JADER	Broad pediatric age category	63,866	reports	2004-2025 descriptive extract	Categorical pediatric age scope; not harmonized to exact age.

Source	Processing stage	Count	Unit	Scope	Interpretation
JADER	Comparable report-level seriousness	Not available	not available	public JADER schema	No FAERS/Canada-comparable public report-level seriousness field; supervised flow stops here.

Panel B. Drug–endpoint–period row accounting

Category	Rows	Unit	Disposition	Interpretation
No FAERS source signal	17,081	drug-endpoint-period rows	Outside supervised analysis	No source-side evidence under the selected signal definition.
Insufficient Canada observability	635	drug-endpoint-period rows	Outside supervised analysis	Canada report support did not meet the observability requirement.
Drug identity outside the primary analysis set	98	drug-endpoint-period rows	Outside supervised analysis	Medicinal-product identity was unsuitable for the primary ingredient-level analysis.
Reserved anaphylaxis support set	18	drug-endpoint-period rows	Separate anaphylaxis analysis	Future-period anaphylaxis rows were reserved before supervised evaluation.
Manufacturer-screened supported rows	147	drug-endpoint-period rows	Supervised benchmark	Positive class in the supervised benchmark.
Canada co-report-absent comparators	481	drug-endpoint-period rows	Supervised benchmark	Comparator class in the supervised benchmark.
Borderline rows (1–2 Canada reports)	155	drug-endpoint-period rows	Withheld uncertainty stratum	Retained for label-sensitivity analysis, not primary fitting.
Ambiguous nonzero Canada signal	20	drug-endpoint-period rows	Withheld uncertainty stratum	Withheld because the Canada evidence was neither positive nor co-report absent.

Supplementary Table S23. Sensitivity of benchmark performance to label and observability definitions. The primary definition required at least three Canada reports, agreement of at least two of three signal families, and a three-report observability threshold. Thirteen specifications produced ten unique label universes because some minimum-report and signal-family combinations were logically equivalent. Model hyperparameters were reused within the original outer folds and were not reselected for individual sensitivity specifications. Intervals are 95% percentile intervals from 2,000 medicinal-product-clustered bootstrap replicates. Specifications that resolve to an identical label universe have identical point estimates; small interval differences reflect independent bootstrap draws.

Definition	Rows	Positives	Conditioned co-report AUPRC	Elastic net AUPRC; paired difference [95% CI]	LightGBM AUPRC; paired difference [95% CI]
Primary definition: at least 3 reports; at least 2 of 3 signal families	628	147	0.753	0.767; +0.014 [-0.019, +0.048]	0.774; +0.022 [-0.022, +0.066]
At least 2 reports; at least 1 of 3 signal families	970	186	0.727	0.718; -0.009 [-0.042, +0.022]	0.768; +0.041 [-0.000, +0.083]
At least 2 reports; at least 2 of 3 signal families	628	147	0.753	0.767; +0.014 [-0.019, +0.050]	0.774; +0.022 [-0.022, +0.066]
At least 2 reports; all 3 signal families	483	125	0.800	0.800; -0.000 [-0.025, +0.026]	0.757; -0.043 [-0.097, +0.005]
At least 3 reports; at least 1 of 3 signal families	970	186	0.727	0.718; -0.009 [-0.039, +0.024]	0.768; +0.041 [+0.000, +0.085]
At least 3 reports; all 3 signal families	483	125	0.800	0.800; -0.000 [-0.025, +0.024]	0.757; -0.043 [-0.096, +0.004]
At least 5 reports; at least 1 of 3 signal families	786	126	0.759	0.745; -0.015 [-0.050, +0.021]	0.788; +0.029 [-0.016, +0.078]
At least 5 reports; at least 2 of 3 signal families	554	106	0.792	0.797; +0.005 [-0.029, +0.040]	0.803; +0.011 [-0.046, +0.063]
At least 5 reports; all 3 signal families	425	90	0.833	0.826; -0.007 [-0.041, +0.025]	0.801; -0.032 [-0.092, +0.016]
Observability sensitivity: at least 1 report	788	147	0.748	0.753; +0.006 [-0.027, +0.038]	0.759; +0.011 [-0.031, +0.055]
Observability sensitivity: at least 5 reports	561	146	0.764	0.789; +0.025 [-0.010, +0.060]	0.777; +0.013 [-0.034, +0.062]
Borderline rows assigned negative	783	147	0.657	0.645; -0.013 [-0.051, +0.023]	0.643; -0.014 [-0.062, +0.043]
Borderline rows assigned positive	783	302	0.705	0.724; +0.019 [-0.003, +0.044]	0.750; +0.045 [+0.000, +0.092]

Supplementary Table S24. Endpoint-stratified comparator performance and paired AUPRC differences. Endpoint-specific estimates are descriptive because several strata contain few supported rows. AUPRC intervals and paired differences use 5,000 medicinal-product-clustered bootstrap replicates. Paired differences are relative to endpoint-conditioned co-reporting and were not adjusted for multiple comparisons.

Endpoint	Comparator	Rows	Positives	AUPRC [95% CI]	AUROC	Brier	ECE	Paired AUPRC difference [95% CI]
Anaphylaxis	Endpoint-conditioned co-reporting	58	34	0.899 [0.792, 0.961]	0.810	0.180	0.220	–
	Elastic-net logistic regression	58	34	0.901 [0.784, 0.963]	0.827	0.166	0.137	+0.003 [-0.047, +0.049]
	Explainable boosting machine	58	34	0.897 [0.787, 0.963]	0.810	0.175	0.153	-0.002 [-0.060, +0.046]
	LightGBM	58	34	0.939 [0.859, 0.983]	0.886	0.142	0.169	+0.040 [+0.002, +0.097]
Seizure	Endpoint-conditioned co-reporting	97	42	0.895 [0.791, 0.962]	0.906	0.122	0.113	–
	Elastic-net logistic regression	97	42	0.867 [0.750, 0.941]	0.877	0.145	0.088	-0.028 [-0.082, +0.012]
	Explainable boosting machine	97	42	0.859 [0.734, 0.939]	0.870	0.144	0.046	-0.036 [-0.100, +0.011]
	LightGBM	97	42	0.865 [0.751, 0.937]	0.861	0.150	0.119	-0.030 [-0.097, +0.027]
Acute kidney injury	Endpoint-conditioned co-reporting	154	32	0.620 [0.396, 0.790]	0.811	0.126	0.077	–
	Elastic-net logistic regression	154	32	0.717 [0.514, 0.855]	0.865	0.101	0.082	+0.097 [-0.002, +0.214]
	Explainable boosting machine	154	32	0.715 [0.512, 0.858]	0.862	0.100	0.052	+0.095 [-0.003, +0.213]
	LightGBM	154	32	0.708 [0.498, 0.852]	0.859	0.106	0.057	+0.088 [-0.007, +0.198]
QT/proarrhythmia	Endpoint-conditioned co-reporting	146	22	0.568 [0.380, 0.739]	0.859	0.097	0.066	–
	Elastic-net logistic regression	146	22	0.600 [0.385, 0.783]	0.869	0.092	0.069	+0.032 [-0.093, +0.161]
	Explainable boosting machine	146	22	0.555 [0.377, 0.771]	0.876	0.093	0.076	-0.013 [-0.145, +0.168]
	LightGBM	146	22	0.593 [0.396, 0.779]	0.845	0.096	0.089	+0.024 [-0.126, +0.179]
Hepatic injury	Endpoint-conditioned co-reporting	173	17	0.472 [0.200, 0.703]	0.730	0.072	0.078	–
	Elastic-net logistic regression	173	17	0.460 [0.191, 0.703]	0.749	0.078	0.067	-0.012 [-0.147, +0.126]
	Explainable boosting machine	173	17	0.455 [0.187, 0.694]	0.752	0.077	0.057	-0.017 [-0.102, +0.056]
	LightGBM	173	17	0.580 [0.291, 0.812]	0.823	0.063	0.056	+0.108 [-0.074, +0.302]

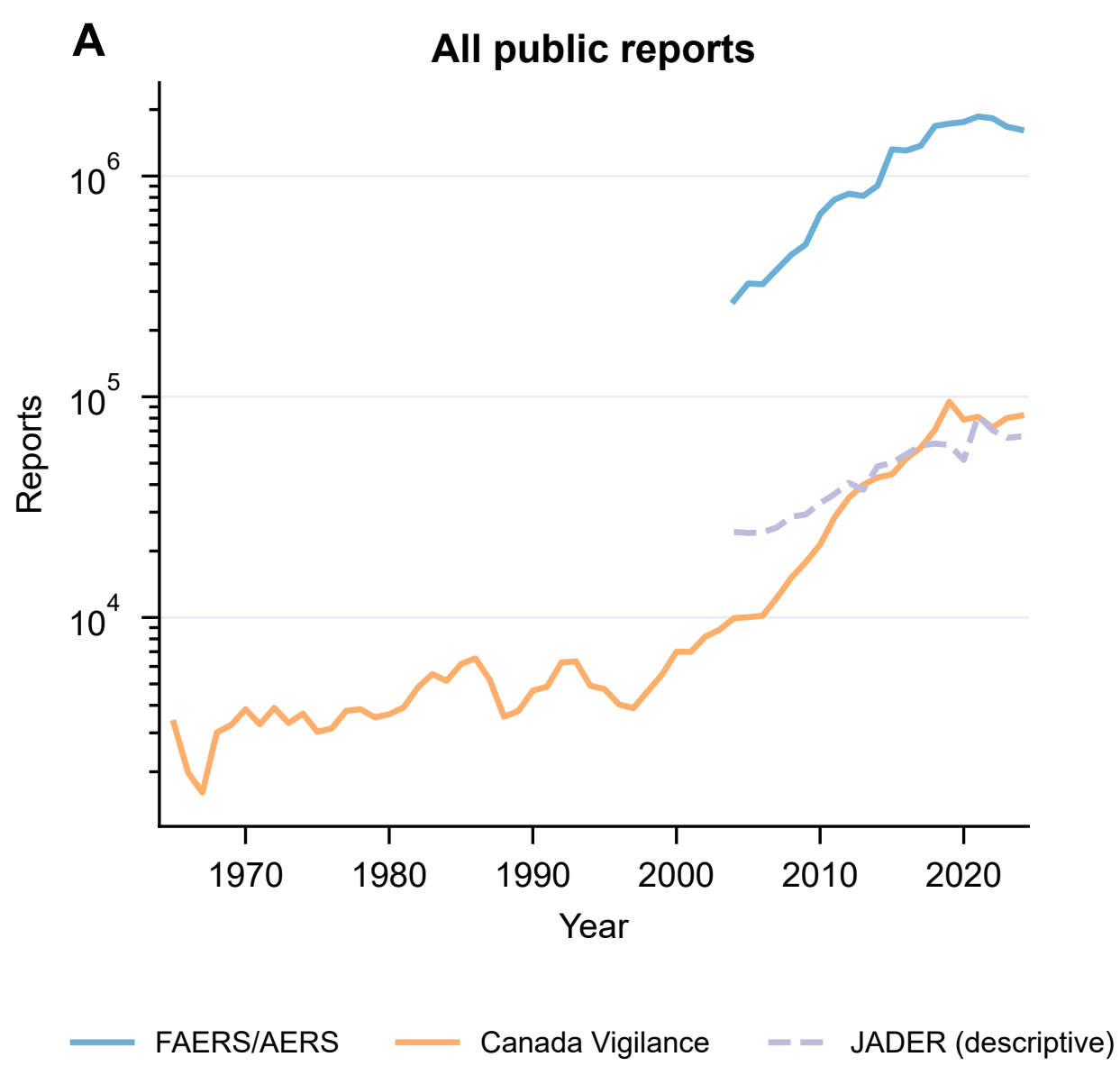

Supplementary Figure S1. Additional public-reporting source landscape. (A) Annual counts of all public reports in FAERS/AERS, Canada Vigilance, and JADER. (B) Annual counts of pediatric serious reports in FAERS/AERS and Canada Vigilance. JADER is shown only in panel A because its public schema lacks a comparable report-level seriousness field.

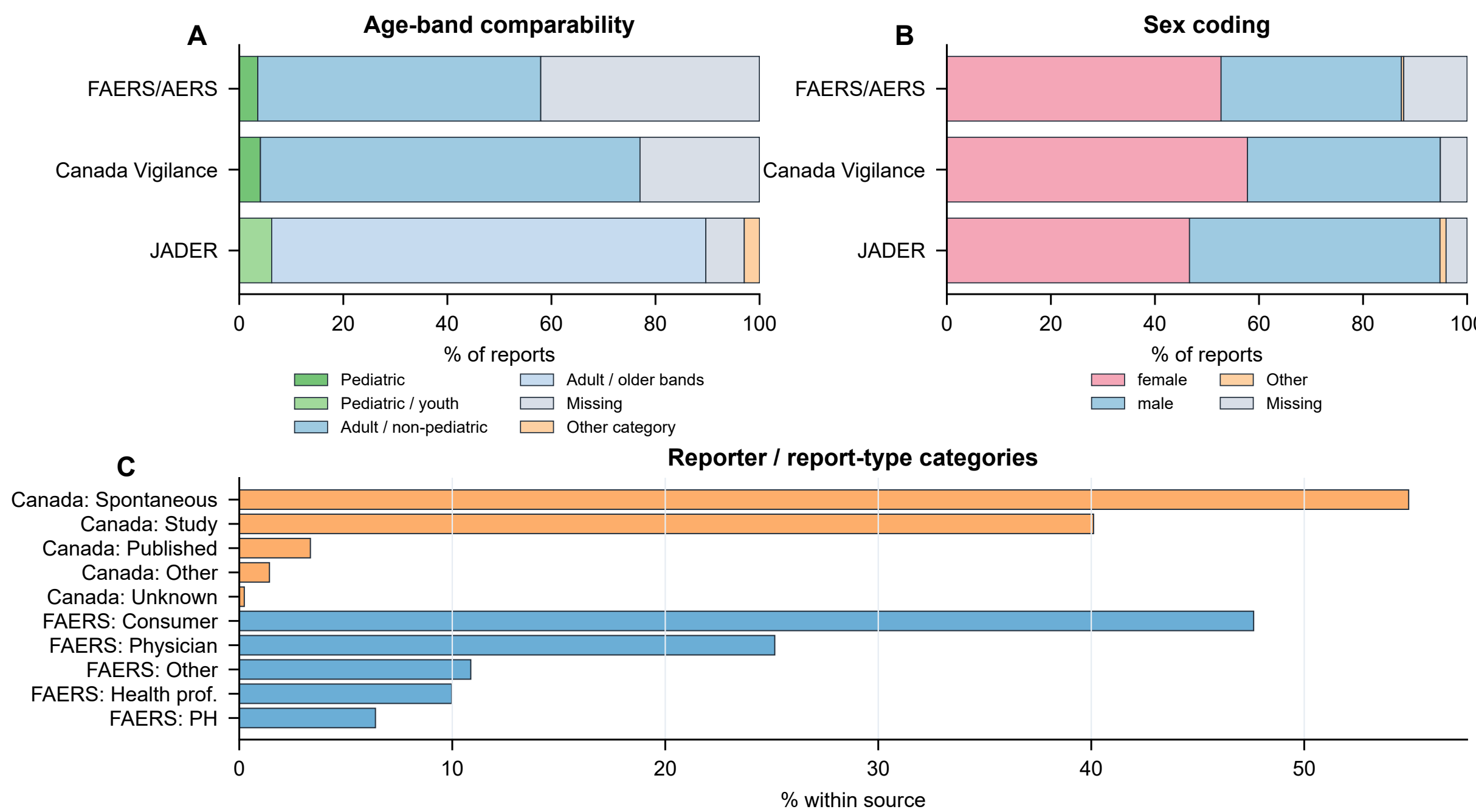

Supplementary Figure S2. Demographic and reporter characteristics by public source. (A) Source-specific proportions across harmonized age-band categories. (B) Source-specific proportions across sex-coding categories. (C) Reporter or report-type categories within FAERS/AERS and Canada Vigilance. These summaries describe reporting composition and public-schema granularity rather than population incidence.

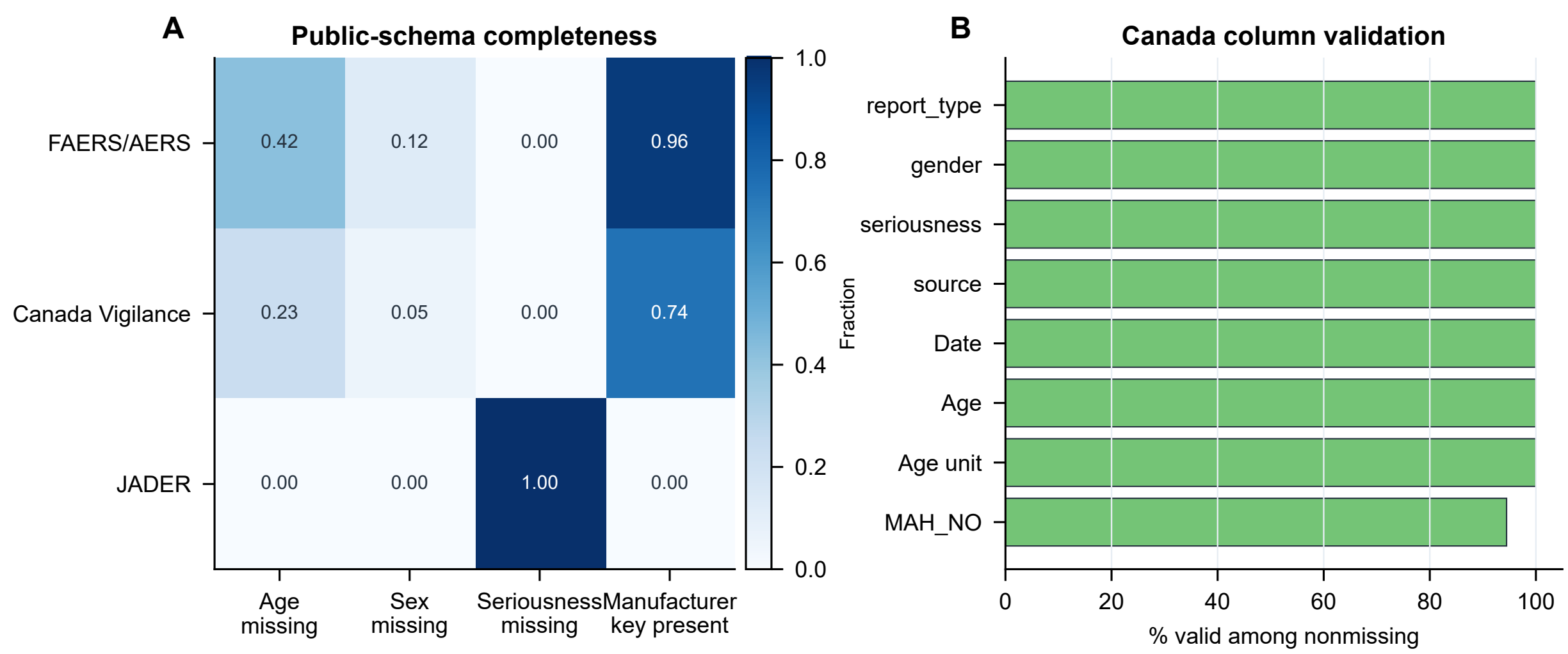

Supplementary Figure S3. Schema completeness and consistency checks. (A) Fractions of reports with missing age, sex, or seriousness information and with a manufacturer key present in each public source. (B) Fractions of non-missing Canada Vigilance values passing field-specific format checks, including the manufacturer authorization holder number (MAH_NO).

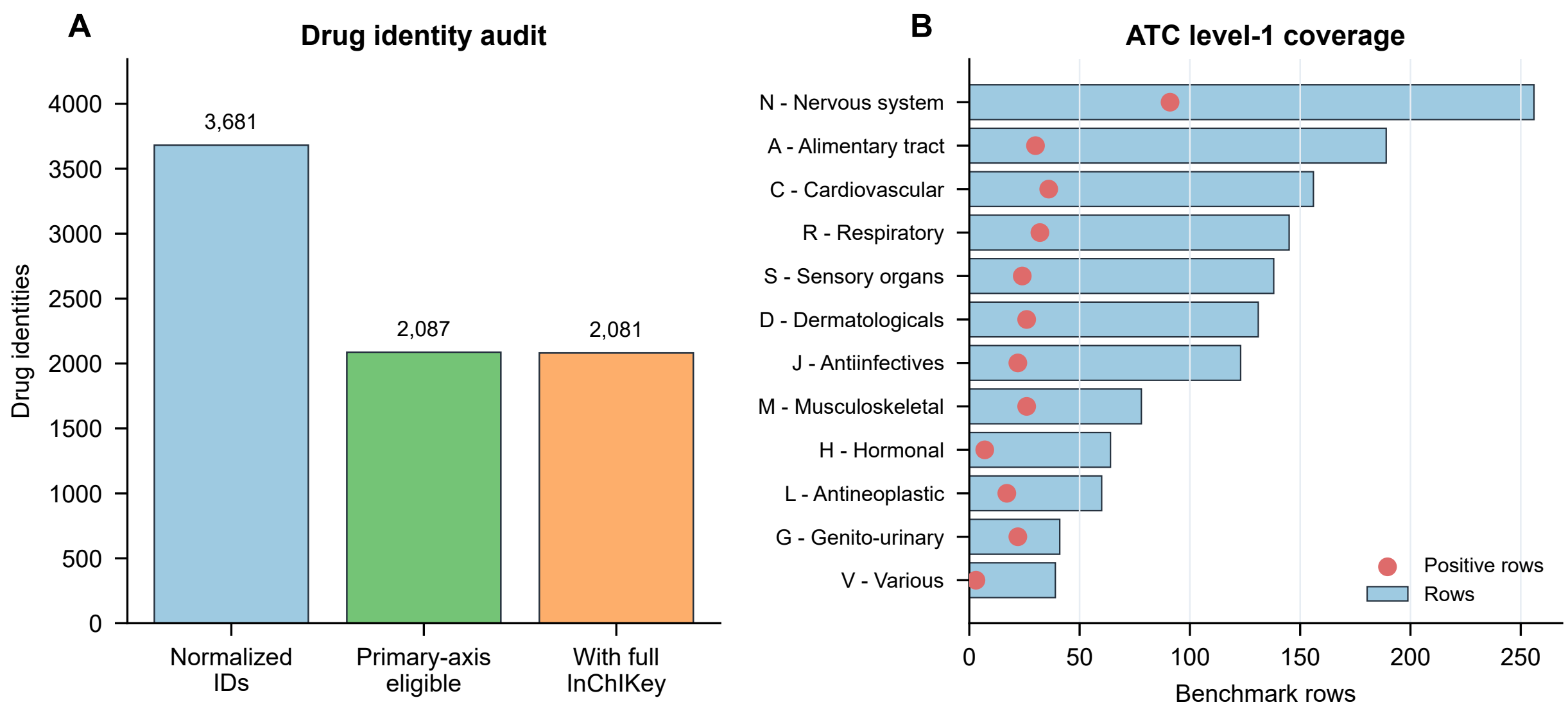

Supplementary Figure S4. Drug-identity and drug-class summaries. (A) Counts of normalized medicinal-product identities, primary-axis-eligible identities, and identities with a full InChIKey. (B) Benchmark rows and positive rows by first-level Anatomical Therapeutic Chemical class.

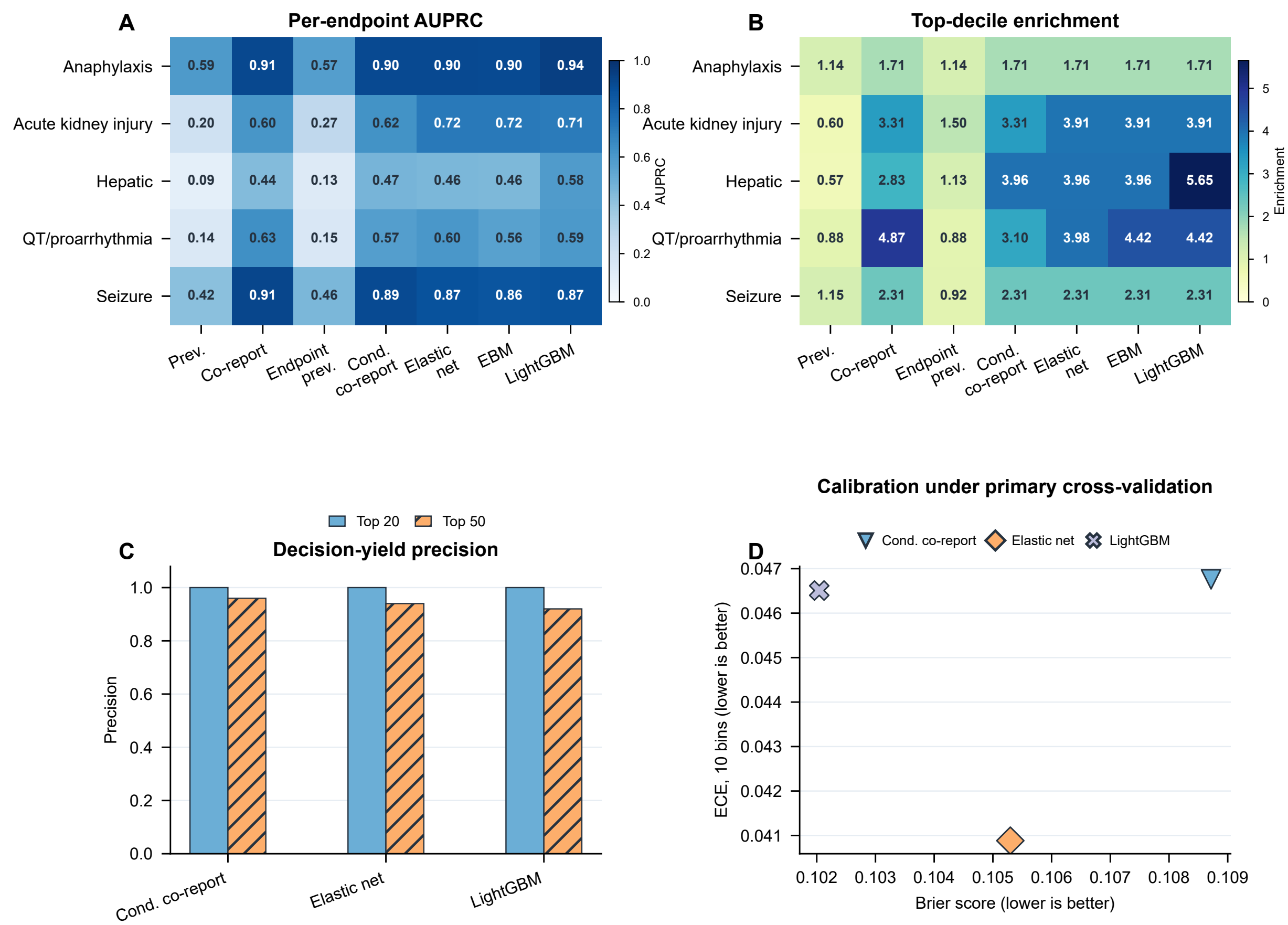

Supplementary Figure S5. Extended benchmark-performance summaries. (A) Per-endpoint AUPRC for the seven supervised benchmark comparators. (B) Per-endpoint top-decile enrichment for the same comparators. Cell values provide a non-color representation of both heatmaps. (C) Precision among the 20 and 50 highest-ranked rows for endpoint-conditioned co-reporting, elastic-net, and LightGBM. (D) Brier score and 10-bin expected calibration error under the primary cross-validation design; marker shape and color identify comparators. Endpoint-stratified results are descriptive because several endpoint strata contain few positives.

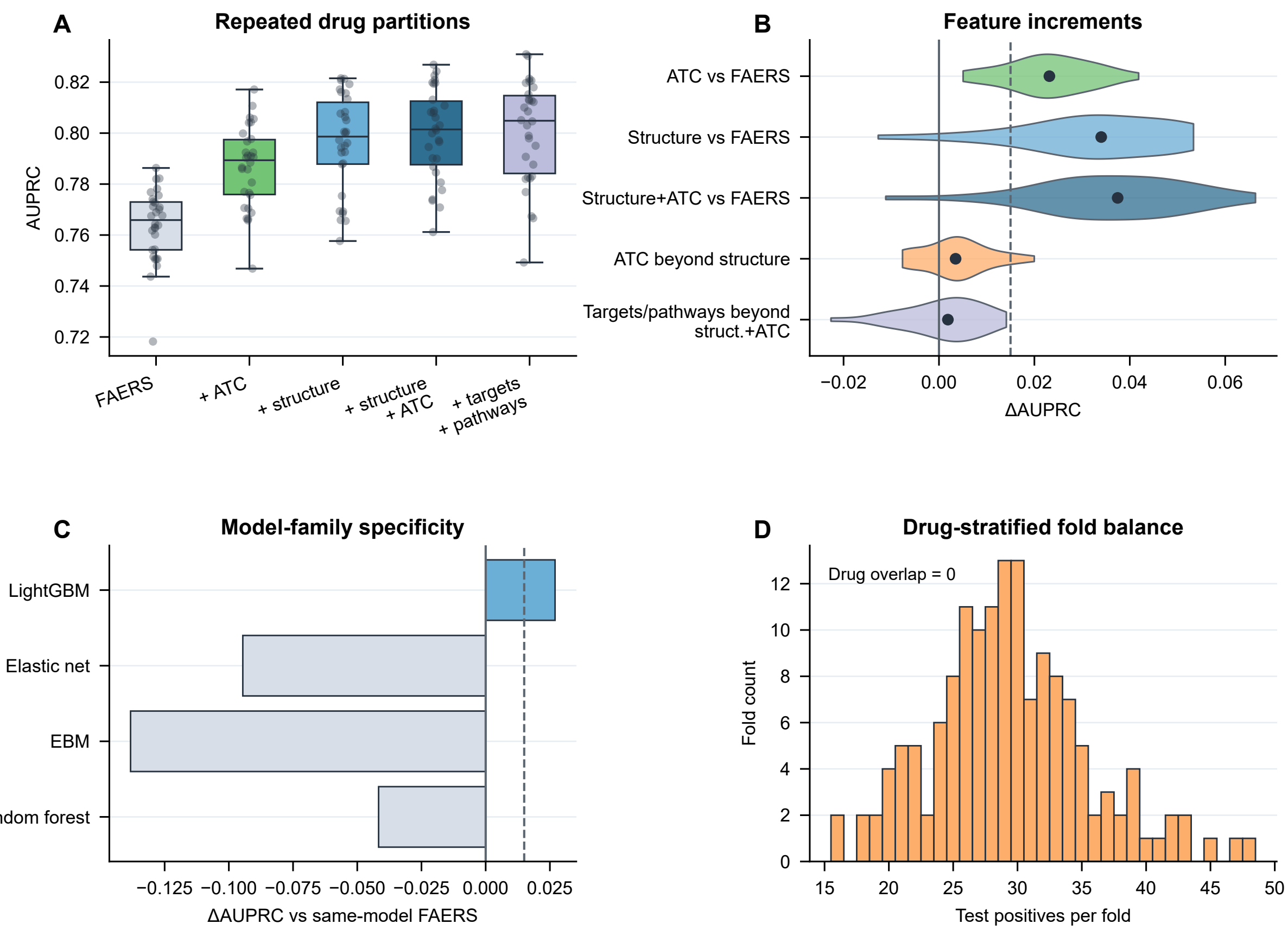

Supplementary Figure S6. Repeated drug-held-out feature-family decomposition. (A) AUPRC distributions across 30 repeated drug-held-out partitions as feature families were added to the full FAERS source-signal baseline. (B) Distributions of the corresponding paired AUPRC increments. (C) Single-holdout AUPRC increments by model family relative to the same-model FAERS baseline. The solid and dashed vertical lines in panels B and C mark zero and the +0.015 exploratory materiality reference, respectively. (D) Distribution of positive-row counts across drug-stratified test folds; no medicinal-product identity crossed between training and test folds.

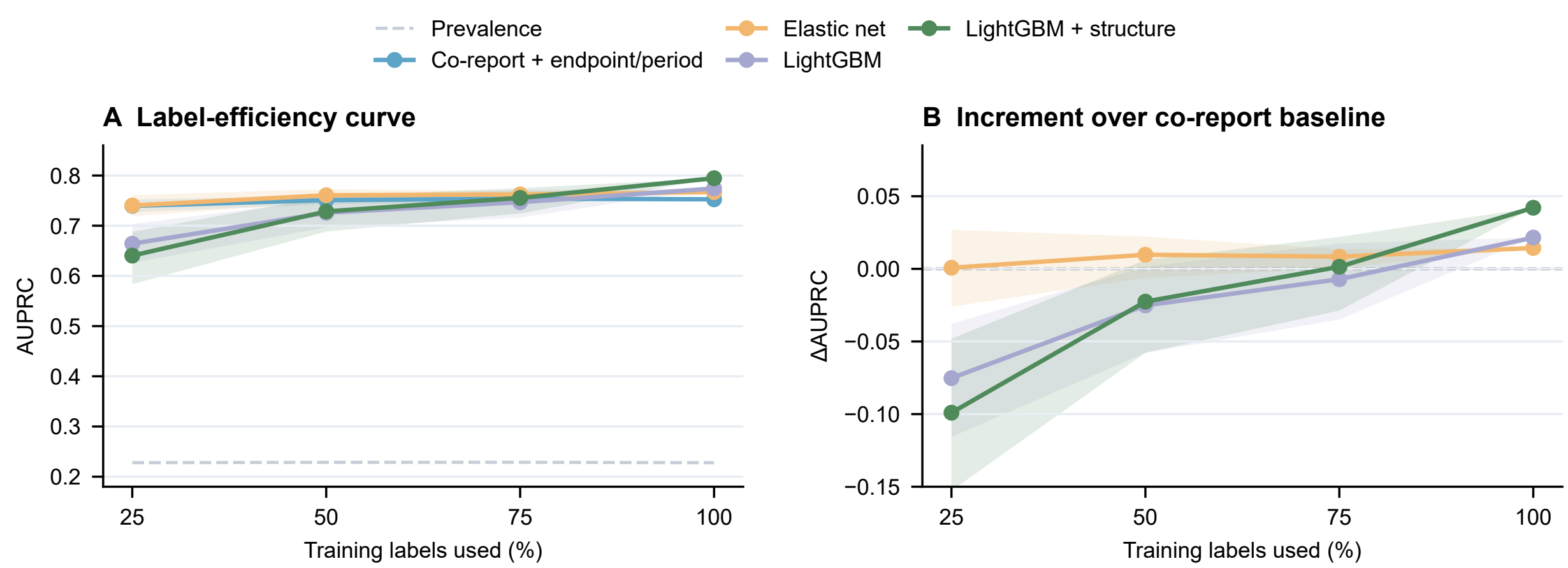

Supplementary Figure S7. Label-efficiency analysis. Training labels were subsampled within each outer training fold to 25%, 50%, 75%, or 100% while retaining the same test folds and model definitions. Panel A shows AUPRC learning curves; shaded bands show the empirical 2.5th–97.5th percentile interval across 30 repeated subsamples for fractions below 100%. Panel B shows the AUPRC increment relative to the endpoint-conditioned co-report comparator.

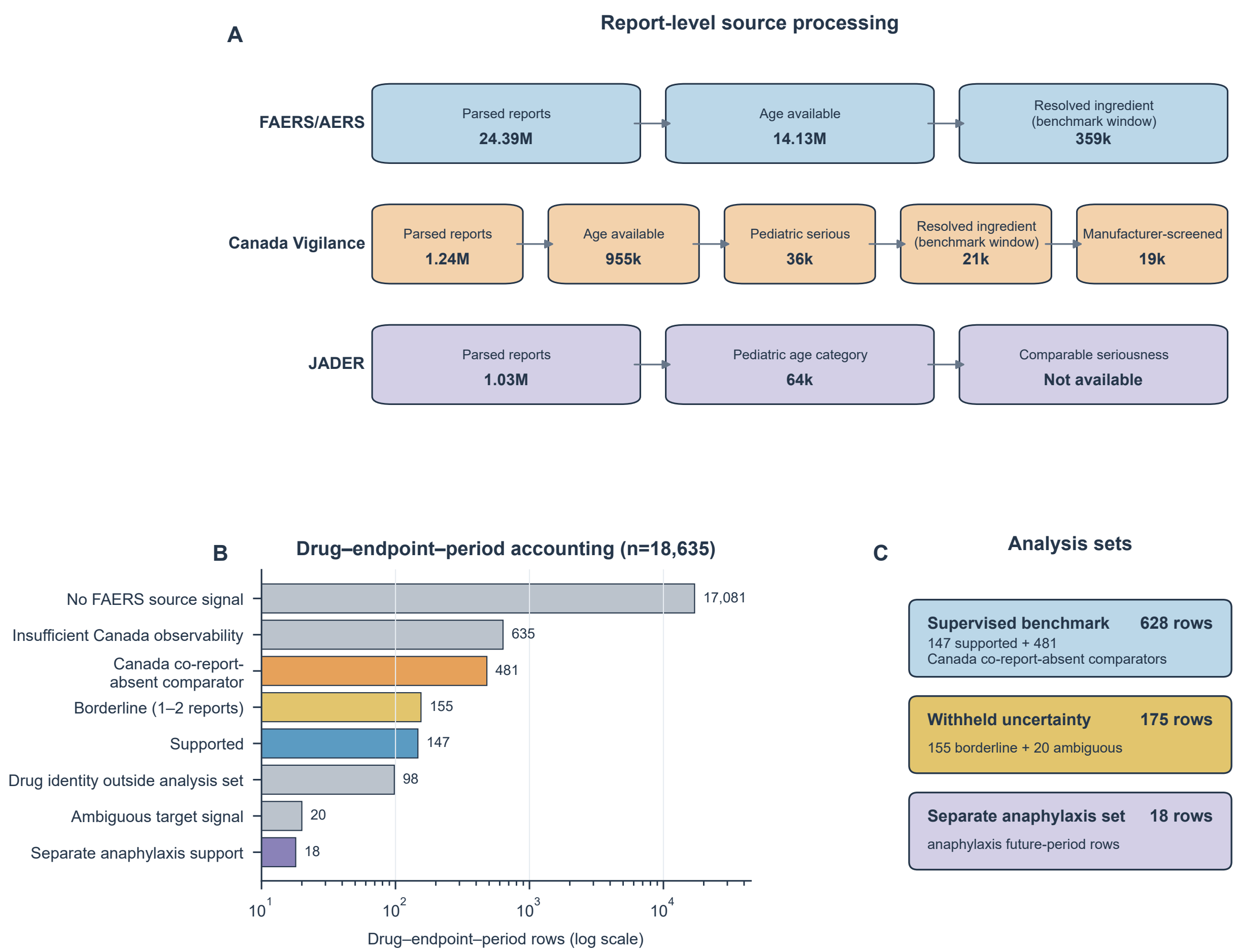

Supplementary Figure S8. Source processing and benchmark-row derivation. (A) Source-specific report processing. Counts are shown with their source-specific scope; the FAERS/AERS ingredient-resolved count and the Canada pre-screen count use the 2014Q3–2024Q4 benchmark period, whereas parsed-source counts cover the full extracts. (B) Accounting for all 18,635 candidate drug–endpoint–period rows. (C) Final supervised, withheld-uncertainty, and separate anaphylaxis analysis sets.

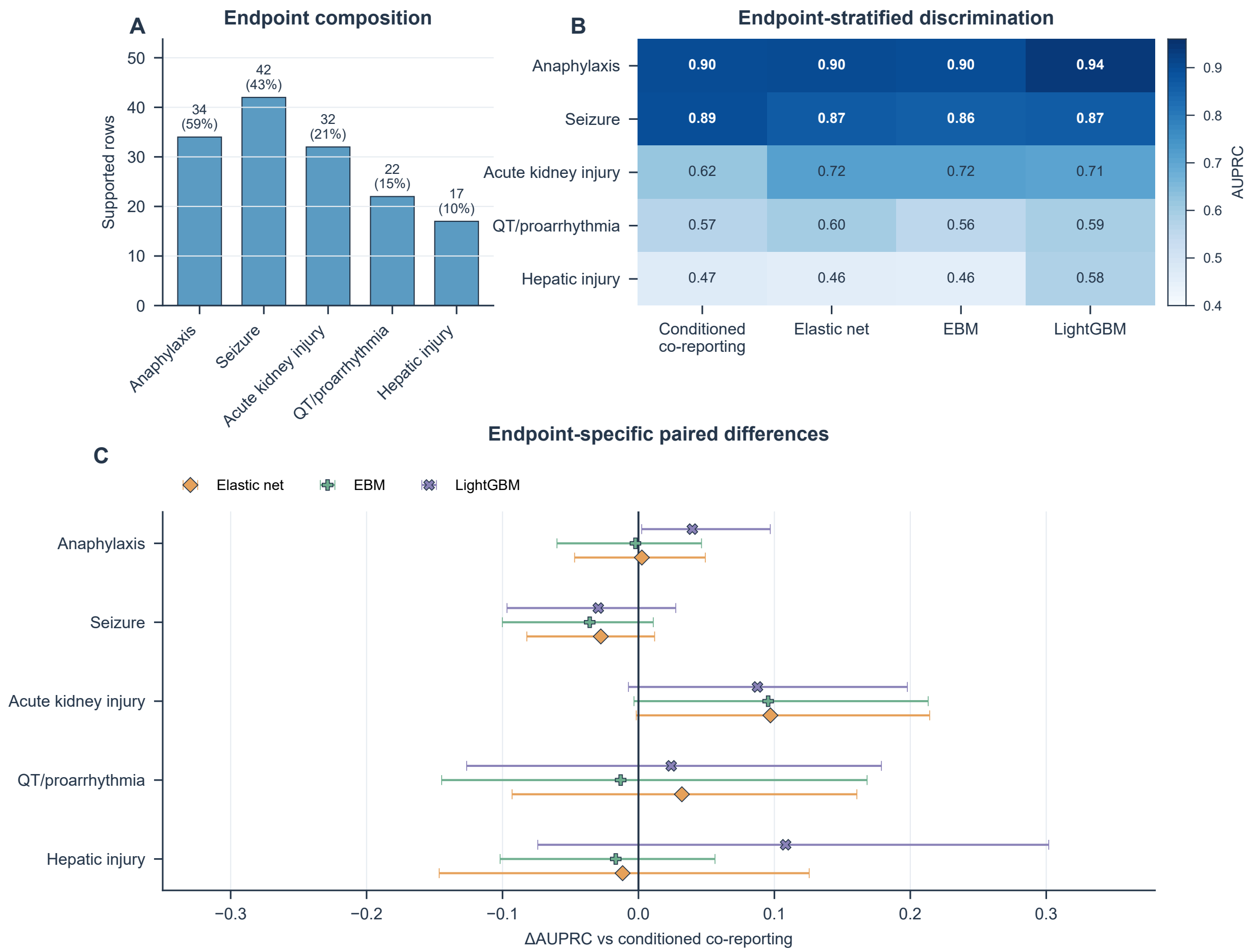

Supplementary Figure S9. Endpoint composition and endpoint-stratified comparator performance. (A) Supported rows and positive fractions by endpoint. (B) Endpoint-specific AUPRC for endpoint-conditioned co-reporting, elastic net, EBM, and LightGBM. (C) Paired AUPRC differences relative to endpoint-conditioned co-reporting, with 95% intervals from 5,000 medicinal-product-clustered bootstrap replicates. Endpoint-specific comparisons were descriptive and were not adjusted for multiple testing.

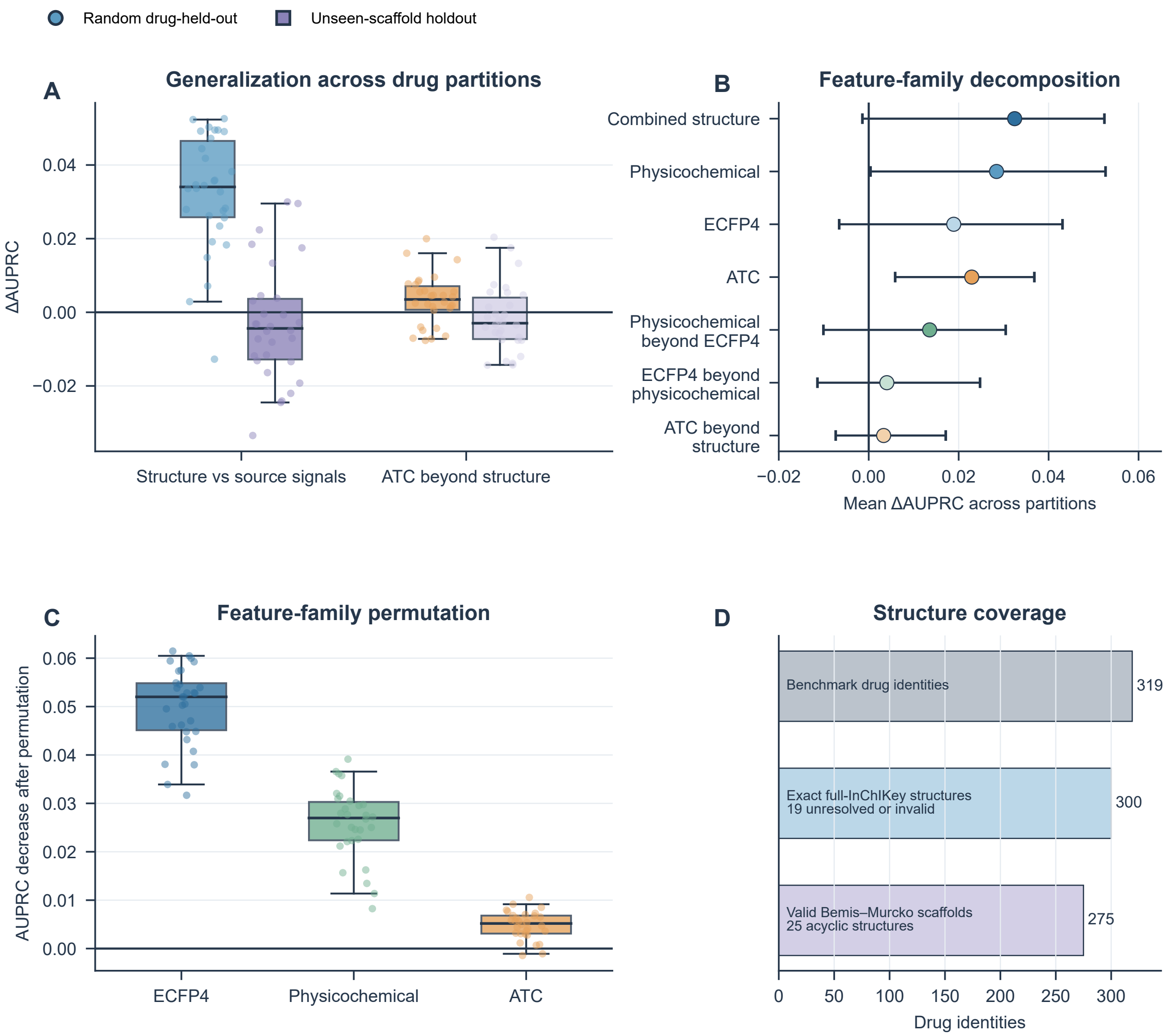

Supplementary Figure S10. Chemical-structure generalization and feature-family attribution. (A) AUPRC differences for structure and for ATC beyond structure across random drug-held-out and unseen-scaffold partitions. (B) Mean feature-family increments across 30 random drug-held-out partitions; horizontal intervals span the 2.5th–97.5th percentiles. (C) AUPRC decrease after within-partition permutation of ECFP4, physicochemical, or ATC features. (D) Coverage of exact structures and valid Bemis–Murcko scaffolds among benchmark medicinal-product identities. The unseen-scaffold analysis included the 275 identities with valid scaffolds; acyclic and unresolved structures are shown in panel D but did not enter that analysis.
